# Activated B cells modulate the maturation of MDSCs via CD36-dependent MHC-II transfer to orchestrate CD4^+^ Th1-dominant antitumor immunity after cryo-thermal therapy

**DOI:** 10.7150/ijbs.115232

**Published:** 2025-08-30

**Authors:** Zelu Zhang, Shicheng Wang, Junjun Wang, Yichen Yao, Yuankai Hao, Yue Lou, Ping Liu, Lisa X. Xu

**Affiliations:** School of Biomedical Engineering and Med-X Research Institute, Shanghai Jiao Tong University, Shanghai, 200000, China.

**Keywords:** Cryo-thermal therapy, B cells, MDSCs, CD36, antitumor immunity, MHC-II

## Abstract

Immunotherapy, particularly immune checkpoint inhibitors (ICIs), has shown great success in treating various cancer types. However, the therapeutic efficacy of ICIs remains unsatisfactory because of the immunosuppressive tumor microenvironment. Cryo-thermal therapy (CTT), a novel tumor ablation approach developed by our laboratory, transforms the tumor immunosuppressive environment into an immunostimulatory environment by activating both innate and adaptive immunity. CTT promotes the differentiation of myeloid-derived suppressor cells (MDSCs) into mature dendritic cells and macrophages, activates antigen-presenting cells and natural killer (NK) cells, and induces Th1-dominant CD4^+^ T-cell-mediated antitumor immunity in numerous highly metastatic tumor models. However, the role of B cells in CTT-induced antitumor immunity remains unclear despite their critical function in adaptive immunity. Here, *in vivo* B-cell depletion with anti-CD20 monoclonal antibodies in multiple tumor models revealed that B cells play a crucial role in suppressing tumor metastasis and extending survival. More interestingly, CTT-activated B cells reprogram MDSCs to a mature phenotype through CD36-dependent major histocompatibility complex class II (MHC-II) transfer, resulting in enhanced Th1-dominant CD4^+^ T-cell responses and CD8^+^ T-cell cytotoxicity. These findings reveal a novel mechanism of B-cell-mediated modulation of the tumor microenvironment and provide insights into enhancing the efficacy of immunotherapy strategies.

## Introduction

Immunotherapy has emerged as a promising cancer treatment. Immune checkpoint inhibitors (ICIs), which activate the immune system to combat the proliferation and spread of cancer cells, have changed the landscape of cancer therapy and have achieved success in numerous types of cancer 1, 2. Nevertheless, the low objective response rate among patients demonstrates the low therapeutic efficacy of ICI therapy 2, which only targets one specific inhibitory mechanism and is unable to fully alleviate the immunosuppressive tumor microenvironment. By reshaping the immunosuppressive tumor microenvironment, cancer cells can evade the host antitumor immune response 3. Immunosuppressive cells such as myeloid-derived suppressor cells (MDSCs), tumor-associated macrophages (TAMs), and regulatory T cells (Tregs) are key players in the tumor immunosuppressive environment, which impairs the activation and cytotoxicity of T cells through a number of mechanisms 4. Thus, there is a growing need to develop more effective immunotherapeutic modalities that are capable of systematically facilitating a microenvironment with increased T-cell infiltration, augmented effector cell activation and reduced MDSC infiltration, thereby promoting a favorable antitumor immune response.

In our previous study, we developed a novel cryo-thermal therapy (CTT) that could completely ablate tumors *in situ* by precooling and subsequent radiofrequency ablation. After CTT, the acute inflammatory response promotes the polarization of M1 macrophages, activates eosinophils and natural killer (NK) cells, and reprograms MDSCs into dendritic cells (DCs) and macrophages 5-8. Moreover, fully mature antigen-presenting cells (APCs) increase the cytotoxicity of CD8^+^ T cells and orchestrate Th1-dominant differentiation of CD4^+^ T cells, resulting in sustained and robust antitumor immunity, leading to long-term survival of tumor-bearing mice 9, 10. Moreover, because of the systematically reversed immunosuppressive environment, CTT further increases the efficacy of cytokine-targeted therapy and adoptive T-cell therapy in a variety of highly metastatic models 11-13. As an integral part of the adaptive immune system, B cells constitute a significant proportion of the cells in the blood and lymphoid tissue, but the role of B cells in CTT-induced antitumor immunity remains unclear.

Increasing evidence has shown that B cells are key mediators of antitumor immunity. In a mouse model, the transfer of agonistic anti-CD40-activated B cells was revealed to suppress tumor growth 14 and induce a potent T-cell antitumor response 15. Moreover, the generation of tumor-specific antibodies by intratumoral B cells plays a role in the effectiveness of immunotherapy 16. Recent studies have revealed that B cells within tumor sites facilitate the clearance of tumor cells via DCs and T cells and are crucial for patient responsiveness to ICI therapy 17-19. Therefore, we investigated the proportion and activation status of B cells and their capacity to respond to CTT, as well as the role of B cells in CTT-induced antitumor immunity.

To investigate the role of B cells in antitumor immunity induced by CTT, B16F10, 4T1 and MC38 mouse tumor models were constructed. B-cell depletion using monoclonal antibodies significantly increased the number of pulmonary metastases and decreased the survival rate of CTT-treated mice, indicating that CTT-induced B cells are crucial for long-term antitumor immunity. RNA sequencing and *in vivo/in vitro* studies further confirmed that CTT-activated B cells restrict the accumulation of splenic MDSCs, modifying the maturation of MDSCs by CD36-dependent transfer of MHC-II molecules and thereby skewing CD4^+^ Th1 cell differentiation to establish a durable antitumor response. Our results provide novel insights into the interaction between B cells and MDSCs in orchestrating systemic antitumor immunity.

## Results

### B cells are essential for the long-term antitumor immunity induced by CTT

**Figure 1:**
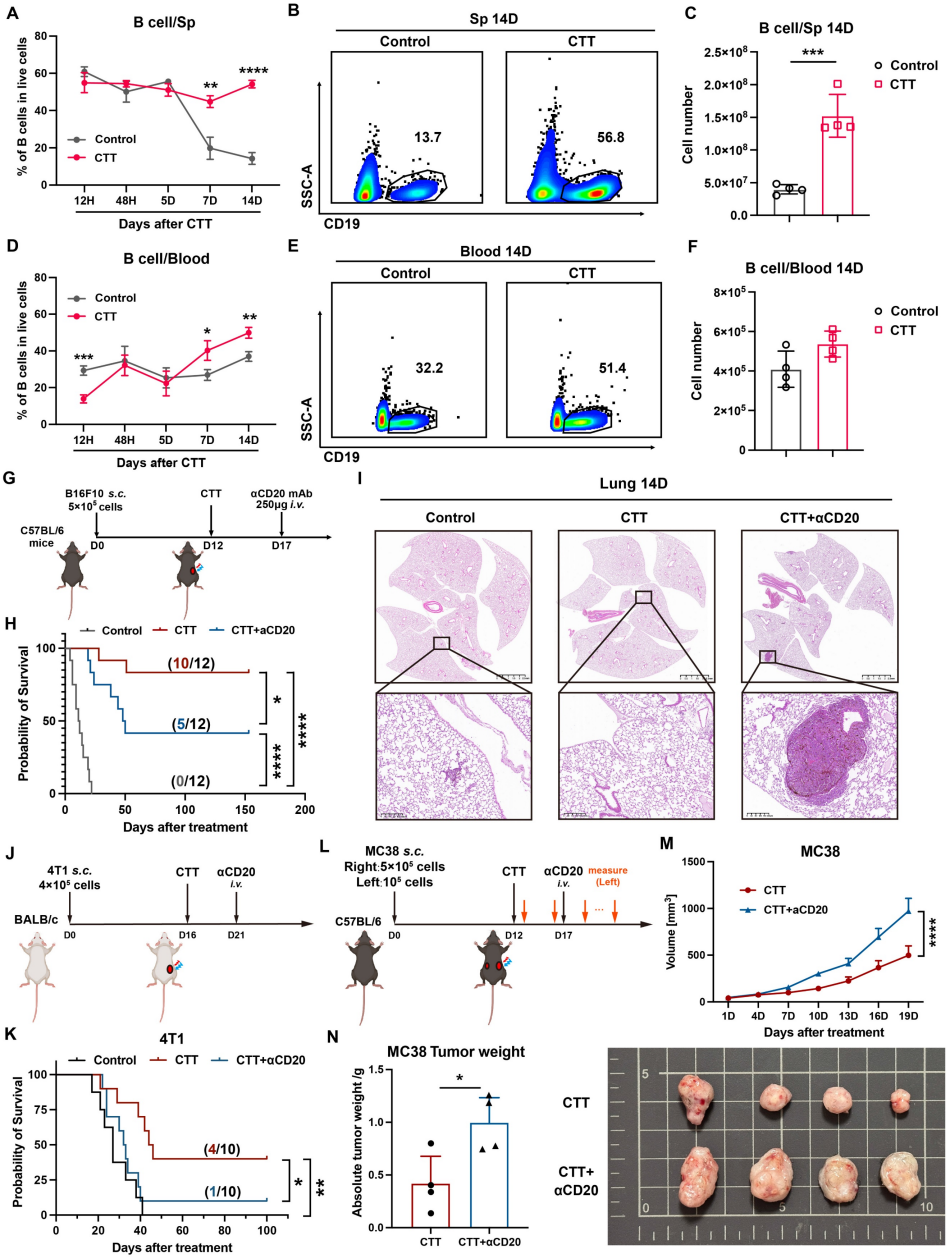
**B cells are essential for the antitumor immunity induced by CTT.** (A) Proportion of B cells in the spleen at different time points after CTT. (B) The proportion of splenic B cells at 14 days after CTT. (C) Absolute number of splenic B cells on day 14 after CTT. (D) The proportions of B cells in the blood at different time points after CTT. (E) Proportion of blood B cells 14 days after CTT. (F) Absolute numbers of B cells in the blood on day 14 after CTT. (G) Schedule of tumor transplantation, CTT, and injection of anti-CD20 monoclonal antibodies in the B16F10 model. (H) Survival rate of B16F10 tumor-bearing mice with anti-CD20 treatment after CTT. (I) The lungs were fixed with paraformaldehyde and collected from different groups on day 14 after CTT and fixed with paraformaldehyde. H&E staining of the lung tissues from the control, CTT and CTT plus αCD20 groups was performed. (J) Schedule of tumor transplantation, CTT, and injection of anti-CD20 monoclonal antibodies in the 4T1 model. (K) Survival rate of 4T1 tumor-bearing mice after CTT with anti-CD20 treatment. (L) Schedule of tumor transplantation, CTT, injection of anti-CD20 monoclonal antibodies and tumor volume measurement in the MC38 model. (M) The tumor (left side) volume of MC38 tumor-bearing mice after CTT with or without anti-CD20 treatment. (N) Tumor weights (left) of MC38 tumor-bearing mice after CTT with or without anti-CD20 treatment on day 20 after CTT. The data in (M) are presented as the means ± SEMs. All other data are presented as the means ± SD. n=4 for each group. *P<0.05, **P<0.01, ***P<0.001, ****P<0.0001. The data in the graphs (C), (F) and (N) were analyzed via two-tailed Student's t tests. Statistical significance was determined using multiple unpaired t tests in (A) and (D) and 2-way ANOVA in (M). In Kaplan‒Meier plots, p values are determined via log-rank (Mantel‒Cox) tests.

To explore the role of B cells in CTT-induced long-term antitumor immunity, we assessed the proportion and number of B cells in peripheral blood and spleen samples from B16F10 model mice at 12 hours and 2, 5, 7, and 14 days after CTT via flow cytometry. In the control (untreated-mice) group, the proportion of B cells in the spleen decreased over time, whereas their proportion in the blood remained relatively unchanged (**Figure 1A, D**). Conversely, after CTT, the percentage of B cells was unchanged in the spleen but increased in the blood (Figure 1A, D). Notably, on day 7 and day 14 after CTT, the percentages of B cells in the blood and spleen in the treatment group were dramatically greater than those in the control group (**Figure 1A-B, D-E**) and were positively correlated with the absolute number of splenic B cells (**Figure 1C**). To determine whether B cells play an indispensable role in CTT-induced antitumor immunity, mice were injected intravenously with anti-CD20 monoclonal antibodies on day 5 after CTT for *in vivo* B-cell depletion (**Figure 1G**). Successful depletion of B cells was confirmed up to 2 weeks after antibody injection via flow cytometry (**Figure S2A**). Consistent with our previous studies, CTT significantly prolonged the survival period and increased the survival rate of B16F10 tumor-bearing mice (**Figure 1H**). However, after B-cell depletion, the survival rate of B16F10 tumor-bearing mice treated with CTT notably decreased from 83.3% to 41.7% (Figure 1H). Moreover, CTT elicited complete protection against lung metastasis, and B-cell depletion after CTT resulted in larger pulmonary tumor nodules than those in the control group (**Figure 1I, S2B**), indicating considerable impairment of the systematic antitumor immunity induced by B-cell depletion after CTT. Similar results were also observed in the 4T1 model, in which B-cell depletion significantly diminished the survival rate of tumor-bearing mice after CTT (**Figure 1J-K**). The MC38 bilateral tumor model also revealed that B cells are essential for the abscopal effect mediated by CTT, as the volume and weight of the left-side tumors significantly increased after CTT with B-cell depletion (**Figure 1L-N**). Overall, these results clearly indicate that B cells play a pivotal role in CTT-induced systematic antitumor immunity to maintain the long-term survival of mice.

### CTT markedly induced B-cell activation

**Figure 2:**
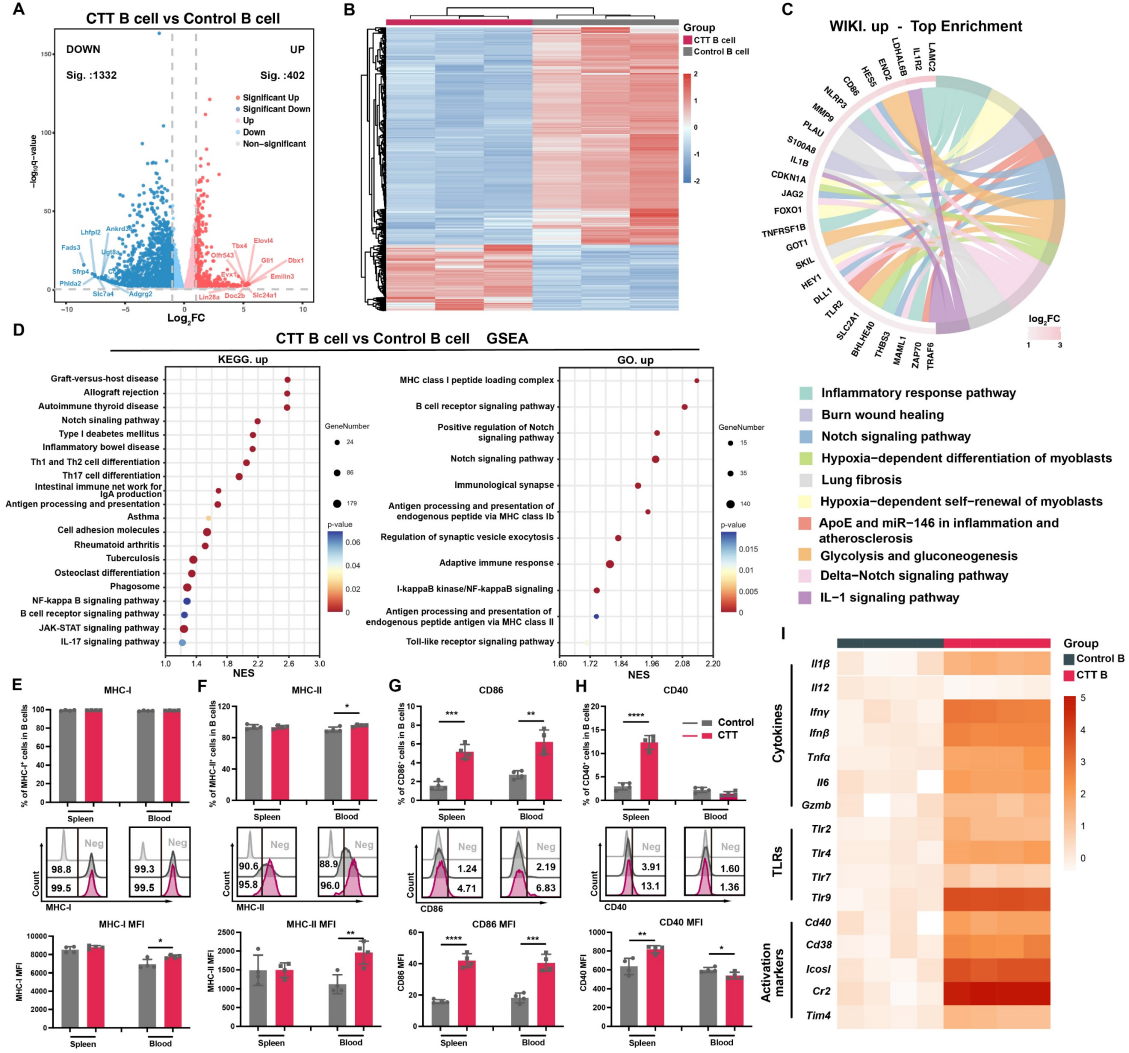
**CTT upregulated the functions of genes encoding antigen processing and presentation and activation markers of B cells.** (A-B) Volcano plot (A) and heatmap (B) showing DEGs between B cells from the control and CTT groups. In the volcano plot, genes with adjusted P values < 0.05 and log_2_FC > 1 values were defined as significantly upregulated signatures, and those with adjusted P values < 0.05 and log_2_FC < -1 values were defined as significantly downregulated signatures. (C) Relationships of the core network with the top 10 Wiki pathways identified via enrichment analysis. (D) Scatter plot of the top 20 enriched KEGG pathways and 10 enriched GO pathways according to GSEA. (E-G) The expression levels of MHC-I (E), MHC-II (F), CD86 (G) and CD40 (H) on B cells. (I) The expression of cytokines, TLRs, and activation markers on B cells on day 14 after CTT was measured via qRT‒PCR. All the data are presented as the means ± SD. n=4 for each group. *P<0.05, **P<0.01, ***P<0.001. The data in the graphs were analyzed via two-tailed Student's t tests.

To investigate the changes in B cells on day 14 after CTT, the gene expression profiles of splenic B cells from CTT-treated and control B16F10 model mice were investigated using 3′ RNA sequencing. Notably, B cells from the CTT group presented significant and distinct transcriptional alterations compared with those from the control group (**Figure 2A, B**). Pathway enrichment analyses revealed marked changes in inflammatory immune response pathways and Notch signaling among the top 10 upregulated Wiki pathways in B cells after CTT (**Figure 2C**). Furthermore, Kyoto Encyclopedia of Genes and Genomes (KEGG) and Gene Ontology (GO) enrichment analyses and gene set enrichment analysis (GSEA) confirmed the activation of the Notch, NFκB, and Toll-like receptor signaling pathways, which are critical for B-cell development, activation, and differentiation after CTT (Figure 2D) 8-10. Moreover, the activation of B-cell receptor (BCR) signaling pathways and antigen processing and presentation indicated that CTT induced a mature phenotype of B cells (**Figure 2D and S3A**). Moreover, GSEA conducted using both the Reactome and KEGG databases revealed a reduction in the cell cycle and DNA replication pathways in B cells after CTT (**Figure S3B-D**). These results reveal the enrichment of pathways involved in activation and antigen processing and presentation in B cells, along with the downregulation of genes involved in the cell cycle after CTT.

Activated B cells can internalize antigens and initiate a T-cell response as antigen*-*presenting cells through the upregulation of major histocompatibility complex (MHC) molecules and costimulatory signals (i.e., CD86 and CD40) 20-24. To investigate whether CTT can induce the expression of these molecules on B cells at the protein level, the expression of MHC-I, MHC-II, CD86 and CD40 on B cells was analyzed on day 14 after CTT via flow cytometry. Because of the high expression of MHC-I and MHC-II on B cells in all the groups, only a slight increase in MHC-II expression on peripheral blood B cells was observed after therapy, but the mean fluorescence intensities (MFIs) of MHC-I on splenic B cells and MHC-II on B cells in the blood were much greater than those in the control group (**Figure 2E, F**). Moreover, substantial increases in the MFI of CD86 and the proportion of CD86^+^ splenic and peripheral blood B cells after CTT, along with increases in the MFI of CD40^+^ splenic B cells and the proportion of CD40^+^ splenic B cells, were observed (**Figure 2G, H**). To further investigate the functional changes in B cells after CTT, splenic B cells from the control and CTT groups were isolated using a MACS isolation kit on day 14 after CTT, and the expression of genes in B cells involved in the inflammatory response and B-cell activation was measured via qRT‒PCR. Compared with those in the control group, high levels of proinflammatory cytokines, such as IL-1β, IFN-γ, IFN-β, TNF-α and IL-6, were observed in B cells after CTT. Additionally, the expression of TLRs (TLR2, TLR4, and TLR9) and activation markers (CD40, CD38, ICOSL, and CR2) on B cells was also upregulated after CTT, indicating that CTT triggered the activation of B cells (**Figure 2I**). Overall, these results indicate that CTT activated B cells, with significant enrichment of differentiation and activation pathways, as well as the upregulation of antigen processing and presentation and immune-stimulating factors.

### B cells orchestrate the differentiation of CD4^+^ T cells and the cytotoxic function of CD8^+^ T cells and promote the maturation of MDSCs after CTT

**Figure 3:**
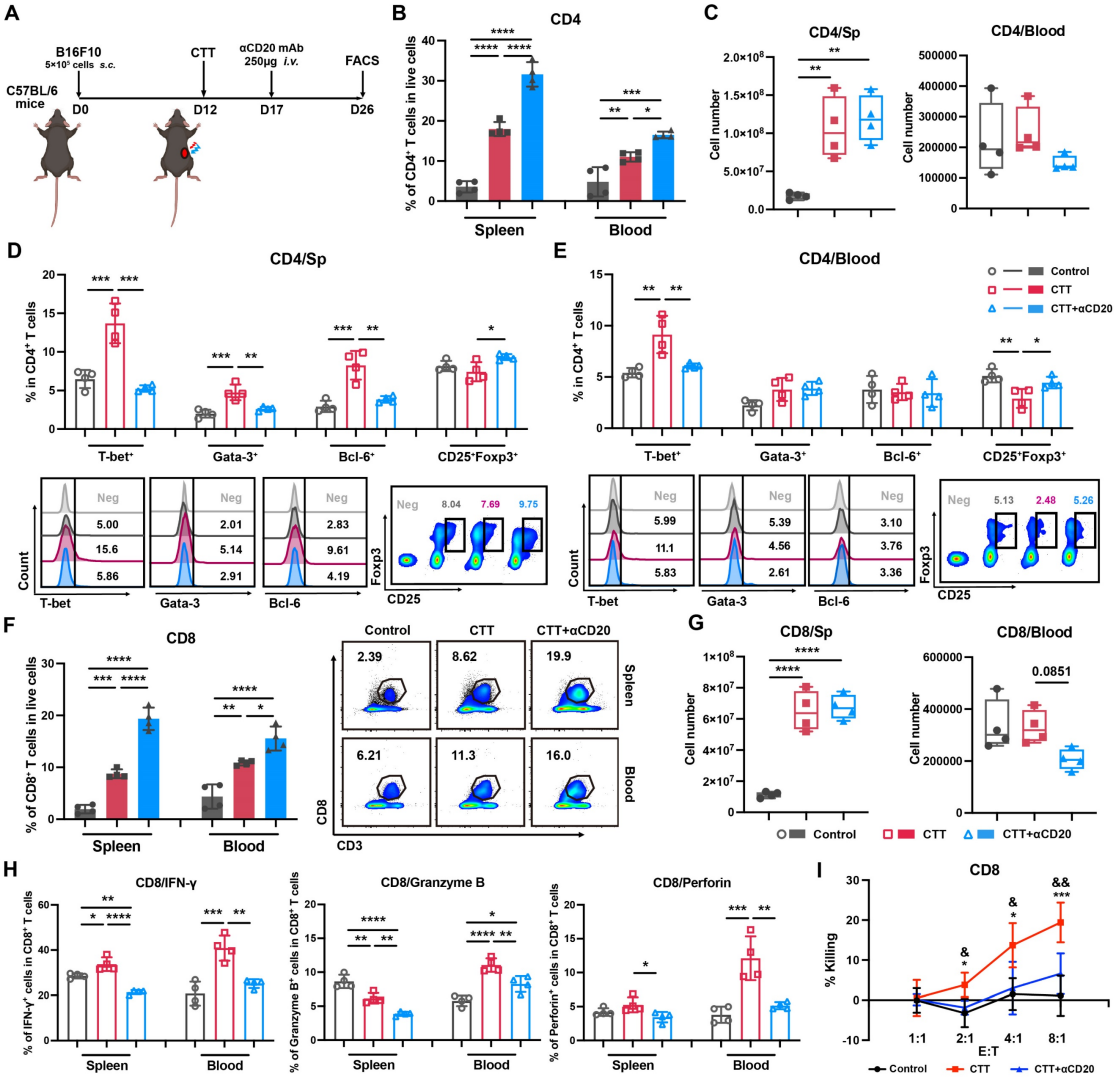
** B-cell depletion inhibits the differentiation of CD4^+^ T cells and the cytotoxic function of CD8^+^ T cells.** (A) Scheme of B16F10 model mice receiving CTT and anti-CD20 antibody injection, as determined via FACS analysis. (B) The percentages of CD4^+^ T cells in the spleen and blood. (C) Absolute numbers of CD4^+^ T cells in the spleen and blood. (D-E) Subsets of CD4^+^ T cells in the spleen (D) and blood (E). (F) The percentages of CD8^+^ T cells in the spleen and blood. (G) Absolute numbers of CD8^+^ T cells in the spleen and blood. (H) IFN-γ, perforin and granzyme-B expression on CD8^+^ T cells in the spleen and blood. (I) Killing assay of splenic CD8^+^ T cells. Briefly, splenic CD8^+^ T cells from different groups were isolated and plated together with calcein-AM-labeled B16F10 melanoma cells at different ratios. The fluorescence intensity of the supernatant was measured after 5 hours of co-culture. Spontaneous calcein release was measured in wells plated with B16F10 cells alone. RIPA lysis buffer was added to the wells containing the target cells alone to measure the maximum calcein release. The percentage of cell killing was calculated by the following formula: 100 × (fluorescence intensity in the experimental well — spontaneous calcein release)/(maximum calcein release — spontaneous calcein release). All the data are presented as the means ± SD. n=4 for each group. *P<0.05, **P<0.01, ***P<0.001, ****P<0.0001. For graph (I), CD8 T cells from the CTT group were compared with those from the control group: *P<0.05, ***P<0.001; CD8^+^ T cells from the CTT group were compared with those from the CTT with B-cell depletion group: &P<0.05, &&P<0.01. The data in the graphs were analyzed via one-way ANOVA.

To further investigate the role of the systemic impact of the B-cell response on antitumor immunity after CTT, an anti-CD20 monoclonal antibody was administered to deplete B cells *in vivo* on day 5 after CTT, and the changes in lymphocyte and myeloid immune cells were analyzed on day 14 after CTT (**Figure 3A**). After CTT, the absolute number of CD4^+^ T cells was significantly greater in the spleen (**Figure 3B**), accompanied by increased proportions of CD4^+^ T cells in the blood and spleen compared with those in the control group (**Figure 3C**). After CTT with B-cell depletion, the percentage of CD4^+^ T cells further increased, indicating the absence of B cells (Figure 3B). However, the absolute number of CD4^+^ T cells did not significantly change in the spleen and tended to decrease in the blood (Figure 3C). Concurrently, subsets of CD4^+^ T cells were analyzed. The results revealed increased percentages of CD4^+^ Th1 cells in the blood and spleen, as well as Th2 and Tfh cells in the spleen, and a significant decrease in the percentage of regulatory T cells (Tregs) in the blood after CTT (**Figure 3D, E**). However, after CTT with B-cell depletion, the proportions of CD4^+^ Th1, Th2 and Tfh cells in the spleen, as well as the proportion of CD4^+^ Th1 cells in the blood, significantly decreased, whereas the percentage of Tregs obviously increased (Figure 3D, E), indicating that B cells were indispensable for CTT-mediated CD4^+^ T-cell differentiation. Furthermore, the absolute number of CD8^+^ T cells in the spleen and the proportions of CD8^+^ T cells in the spleen and blood were significantly greater in the CTT group than that in the control group (**Figure 3F, G**). However, after CTT with B-cell depletion, despite the increased percentages of CD8^+^ T cells in both the spleen and blood, there were no evident changes in the absolute number of CD8^+^ T cells in the spleen, and a declining trend in the blood was observed (Figure 3G). Notably, the expression levels of cytotoxic molecules, including IFN-γ, Granzyme-B and perforin, in CD8^+^ T cells were thoroughly diminished after CTT with B-cell depletion (**Figure 3H**). Moreover, an *in vitro* CD8^+^ T-cell cytotoxicity assay further confirmed that the potent tumor-killing capacity of CD8^+^ T cells was significantly impaired after CTT with B-cell depletion (**Figure 3I**). In summary, these results indicate that B cells are critical for driving the differentiation of CD4^+^ T cells and preserving the cytotoxic function of CD8^+^ T cells after CTT.

The changes in NK cells after CTT with B-cell depletion were also analyzed. The percentages of NK cells in the spleen and blood, along with the absolute number in the spleen, obviously increased after CTT (**Figure S4A-B**). However, the absolute number of NK cells significantly decreased in the blood after CTT with B-cell depletion (Figure **S4B**). The expression of cytotoxic molecules (IFN-γ and perforin) in NK cells was increased after CTT, which indicated that CTT activated NK cells in both the spleen and blood (**Figure S4C**). Nonetheless, B-cell depletion did not significantly change the expression of cytotoxic molecules in NK cells after CTT (Figure S4C) and had a tumor-killing capacity similar to that of the CTT group (**Figure S4D**). These results suggest that B-cell depletion decreased the number of NK cells in peripheral blood.

**Figure 4:**
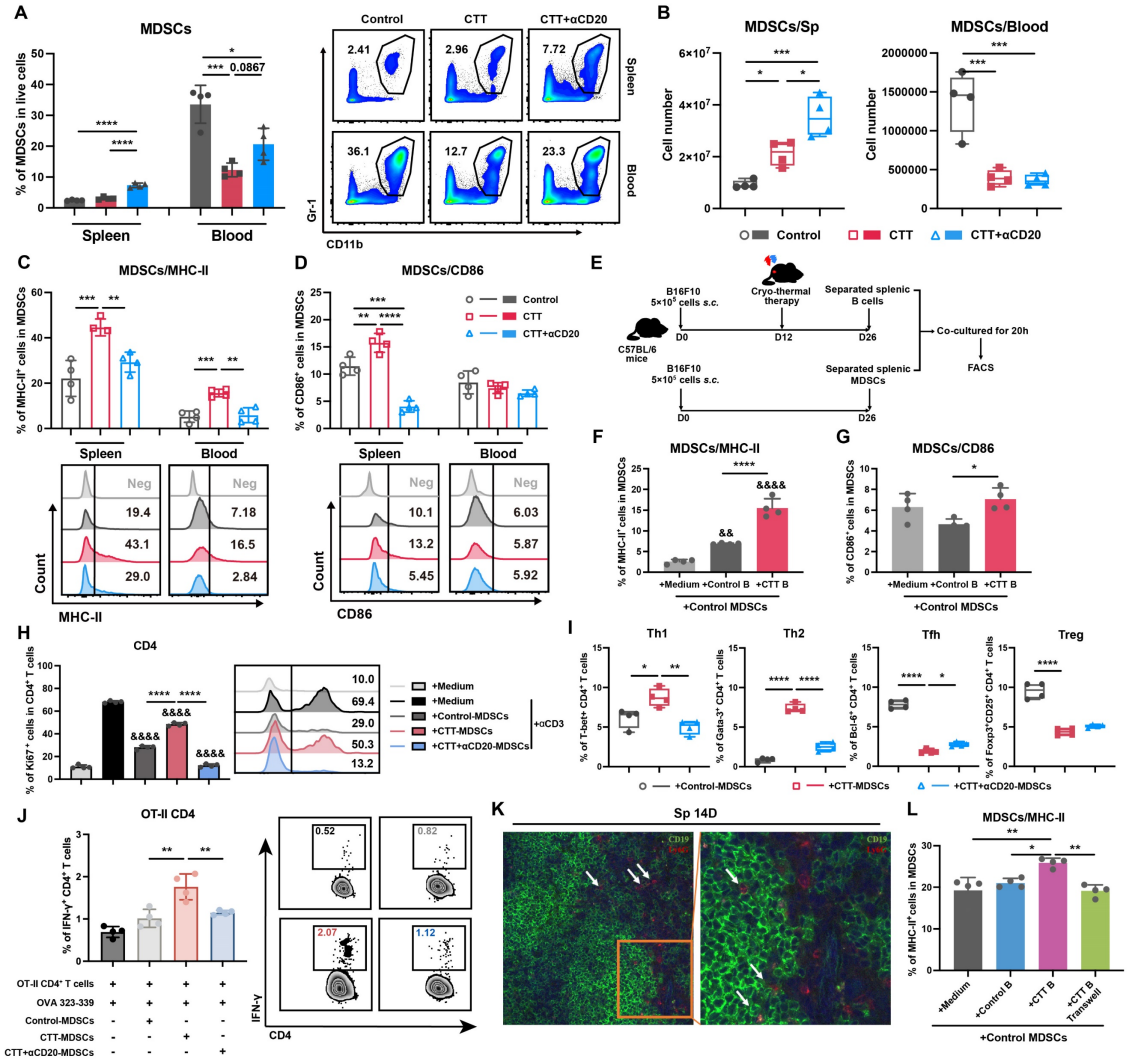
** B cells promote the antigen-presenting phenotype of MDSCs in a soluble molecule-independent manner.** (A-B) Proportion (A) and number (B) of MDSCs in the spleen and blood 14 days after CTT. (C-D) The expression of MHC-II (C) and CD86 (D) on MDSCs. (E-G) B cells from the control group and CTT group were co-cultured with MDSCs from the control group, and the expression of MHC-II (F) and CD86 (G) molecules was analyzed after 20 hours by FACS. (H) Ki67 expression in CD4^+^ T cells cultured with MDSCs from the control, CTT or CTT with anti-CD20 groups for 72 h; E:T = 2:1. (I) MDSCs from the control, CTT or CTT with anti-CD20 groups were separately cultured with CD4^+^ T cells from the control group, and the subsets of CD4^+^ T cells were tested after 24 hours. (J) IFN-γ expression in OT-II CD4^+^ T cells after co-culture with MDSCs from the control, CTT or CTT+anti-CD20 groups in the presence of the OVA 323-339 peptide (10 µg/ml) for 18 h. (K) Immunofluorescence staining with APC-conjugated anti-mouse Ly6G, rabbit anti-mouse CD19 and Alexa Fluor 555-conjugated anti-rabbit IgG antibodies. (L) The expression of MHC-II molecules on MDSCs. All the data are presented as the means ± SD. n=4 for each group. *P<0.05, **P<0.01, ***P<0.001, ****P<0.0001. For graph (H), other groups were compared with the medium plus αCD3 group: &&&&P<0.0001. The data in the graphs were analyzed via one-way ANOVA.

To comprehensively investigate the potential changes in myeloid cells after CTT with B-cell depletion, the changes in MDSCs, DCs, and macrophages were analyzed via flow cytometry. On day 14 after CTT, both the percentage and absolute number of MDSCs were decreased in the blood (**Figure 4A, B**). Moreover, the expression of MHC-II and CD86 on splenic MDSCs and the expression of MHC-II on MDSCs from blood were obviously increased (**Figure 4C, D**), which is consistent with our previous studies 10, 25. However, both the percentage and the absolute number of splenic MDSCs were increased after CTT with B-cell depletion (Figure 4A, B). Furthermore, the expression of MHC-II on MDSCs from the spleen and blood, as well as the expression of CD86 on splenic MDSCs (both G-MDSCs and M-MDSCs), was markedly decreased after CTT with B-cell depletion (**Figure 4C-D, S5A-B**). Moreover, CTT with B-cell depletion led to decreased expression of CD86 on DCs and macrophages compared with that after CTT (**Figure S6A-D**). The results reveal that, after CTT, B cells decrease the accumulation of MDSCs in the peripheral blood and promote the maturation of MDSCs.

To investigate the applicability of the immunostimulatory role of B cells in a wider range of tumor models, changes in other immune cells were analyzed in 4T1 and MC38 bilateral models. B-cell depletion significantly promoted the expansion of MDSCs, inhibited the expression of MHC-II on both G- and M-MDSCs, and systematically diminished Th1-dominant CD4^+^ T-cell differentiation in both models after CTT (**Figure S7A-H, S8A-I**). Moreover, the cytotoxic molecule expression of CD8^+^ T cells was impaired after CTT with B-cell depletion in MC38 bilateral models (**Figure S8J**). These results indicate that the immunostimulatory effects of CTT-induced B cells are applicable across tumor models.

Taken together, these data indicate that B cells are essential for orchestrating CD4^+^ Th1-dominant differentiation after CTT, amplifying the cytotoxic function of CD8^+^ T cells, increasing the number of circulating NK cells, and facilitating the maturation of MDSCs.

### Activated B cells regulate T-cell differentiation and effector function after CTT by modulating MDSCs

Our previous study substantiated the essential role of Th1-dominant T cells in orchestrating sustained antitumor immunity after CTT 10. Given the pivotal role of B cells in T-cell activation, we wondered whether B cells could directly regulate the Th1-dominant immune response. Therefore, the splenic B cells isolated from each group on day 14 after CTT were co-cultured with splenic CD4^+^ T cells or CD8^+^ T cells from the control group for 24 h *in vitro*, and the subtypes of CD4^+^ T cells and the expression of cytotoxic effector molecules in CD8^+^ T cells were examined via flow cytometry (**Figure S9A**). Surprisingly, compared with B cells from the control group, B cells from the CTT group did not promote the Th1-dominant CD4^+^ T-cell differentiation or the expression of effector molecules in CD8^+^ T cells after co-culture, which was inconsistent with the *in vivo* results (**Figure S9B-C**). These results suggest that B cells do not directly modulate the T-cell immune response at the late stage (on day 14) after CTT.

Our previous investigation revealed that mature MDSCs exhibit an attenuated immunosuppressive capacity, manifested by a reduction in the inhibition of T-cell proliferation and the Th1 polarization of CD4^+^ T cells 8. As B cells facilitate the maturation of MDSCs *in vivo*, we sought to determine whether B cells could modulate the T-cell immune response by reprogramming MDSCs. To investigate whether B cells could drive the maturation of MDSCs after CTT, splenic MDSCs from the control group were isolated and co-cultured with splenic B cells from the control or CTT group (**Figure 4E**). As shown in **Figure 4F**, the expression of MHC-II on MDSCs was markedly increased by approximately 2.5-fold when B cells from the CTT group were cultured compared with those from the control group. Furthermore, the expression of CD86 on MDSCs cultured with B cells from the CTT group was substantially greater than that on those cultured with B cells from the control group (**Figure 4G**). These results suggest that B cells directly promote the maturation of MDSCs after CTT.

To explore whether B-cell-exposed MDSCs regulate the T-cell immune response, splenic MDSCs from the control, CTT, or CTT with B-cell depletion groups were isolated on day 14 after CTT and co-cultured with splenic T cells from the control group. The proliferation and subtypes of T cells were subsequently investigated via FACS after co-culture. The results revealed that the proliferation of both CD4^+^ and CD8^+^ T cells co-cultured with MDSCs from the CTT with B-cell depletion group was dramatically suppressed compared with that of those co-cultured with MDSCs from the CTT or control groups (**Figure 4H, S10**), indicating that activated B cells induced by CTT abrogated the inhibitory effects of MDSCs on T-cell proliferation. Moreover, compared with those from the control group, MDSCs from the CTT group facilitated the differentiation of CD4^+^ T cells into Th1 and Th2 cells while concurrently inhibiting the differentiation of CD4^+^ T cells into Tfh cells and Tregs (**Figure 4I**). However, when co-cultured with MDSCs from the CTT with B-cell depletion group, the percentages of CD4^+^ Th1 and Th2 cells were significantly decreased, whereas the percentages of CD4^+^ Tfh cells were increased compared with those in the group co-cultured with MDSCs from the CTT group (Figure 4I). Additionally, an antigen presentation assay confirmed that compared with those from the control group, CTT-induced MDSCs could present antigens to CD4^+^ T cells from OT-II transgenic mice (**Figure 4J**). However, the antigen-presenting capacity of MDSCs was significantly abrogated after CTT with B-cell depletion (Figure 4J). These results indicate that after CTT, activated B cells diminish the immunosuppressive effect of MDSCs on T-cell proliferation and facilitate the maturation and antigen-presenting capability of MDSCs, leading to the Th1-dominant differentiation of CD4^+^ T cells.

### CTT facilitates the integration of functional MHC-II from B cells into the membrane surfaces of MDSCs

To further elucidate the mechanism by which B cells orchestrate the maturation of MDSCs, immunofluorescence staining was conducted to analyze the cellular localization of B cells and MDSCs in the spleen on day 14 after CTT. An anti-mouse Ly6G antibody was utilized to label G-MDSCs, which are more prevalent than Ly6C^+^ MDSCs (M-MDSCs) in the spleen. The results revealed the colocalization of G-MDSCs with B cells in the spleen (**Figure 4K**). Moreover, when we used 0.4-μm Transwell chambers to physically separate CTT-induced B cells and MDSCs, the expression of MHC-II and CD86 on MDSCs was comprehensively downregulated compared with that in the co-culture group (**Figure 4L**,** S11A-D**). Similar results were also observed in the 4T1 model (**Figure S11E-H**). These results suggest that, after CTT, B cells promote the maturation of MDSCs independent of soluble factors.

**Figure 5:**
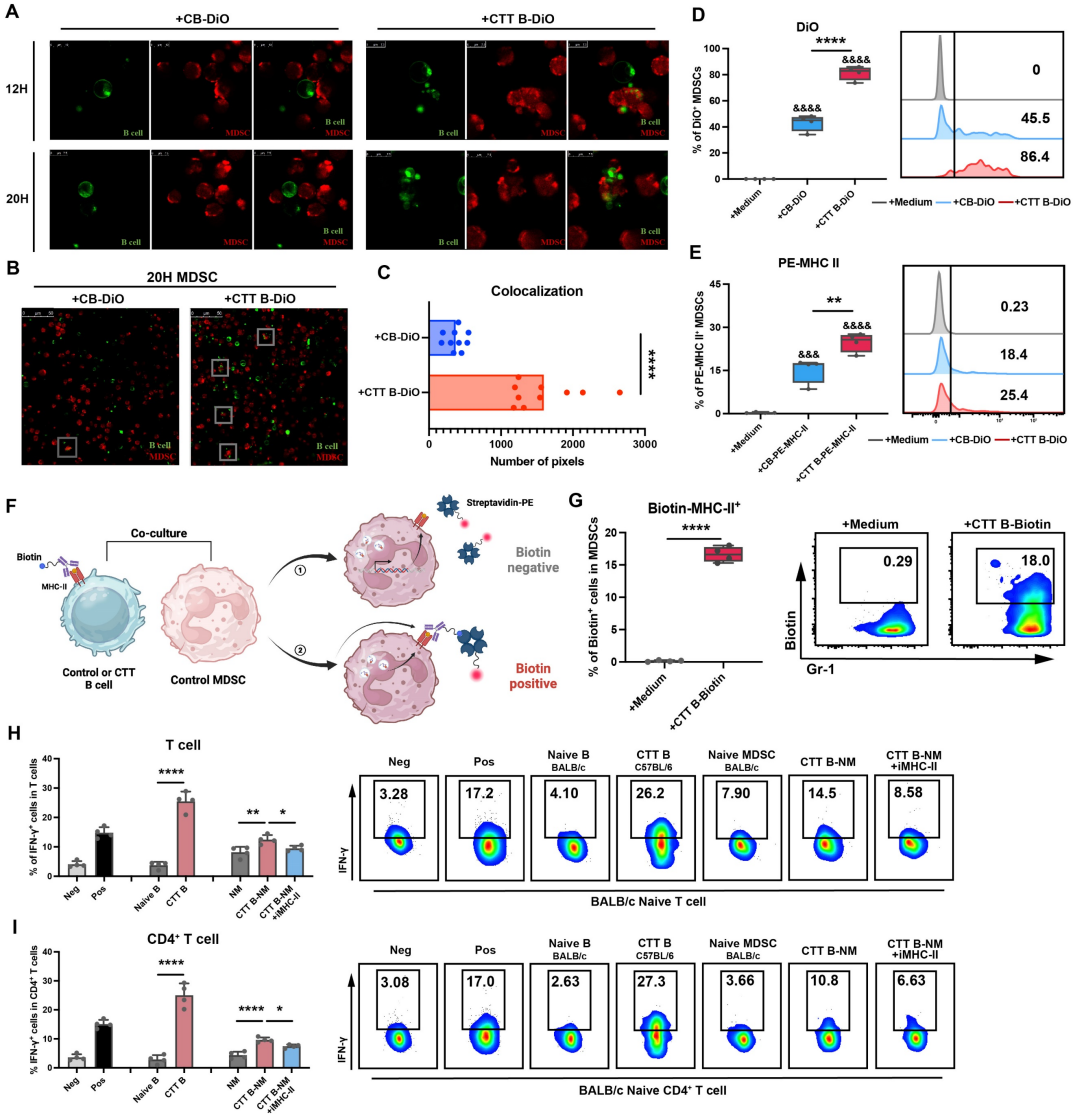
** MDSCs integrate functionally configured MHC-II molecules from B cells after CTT.** (A) Zoomed-in images of MDSCs cultured with control B cells (CB) or CTT B cells after 12 or 20 hours. MDSCs were labeled with CellTrace™ Far Red (Red), and B cells were labeled with DiO (Green). (B) A representative image of MDSCs cultured with different B cells. (C) The colocalization pixels of red and green fluorescence were analyzed by ImageJ in 10 different views of each group. (D) The percentage of DiO^+^ MDSCs among the groups after 20 hours of co-culture was measured by FACS. (E) The percentage of PE^+^ MDSCs among the groups after 20 hours of co-culture was measured by FACS. (F) Schematic of the location of B-cell-derived MHC-II on MDSCs. (G) MDSCs from the control group were cultured with CTT B cells labeled with Biotin-MHC-II antibodies for 20 h, and streptavidin-PE was used to detect the Biotin^+^ MDSCs. (H-I) The functional activity of CTT B-cell (C57BL/6 background)-educated naïve MDSCs (BALB/c background) (CTT B-NM) was determined via the allogeneic MLR assay, with or without an anti-mouse MHC-II antibody, with BALB/c mouse T lymphocytes used as responder cells. Neg: T cells or CD4^+^ T cells cultured alone in complete medium. Pos: T cells or CD4^+^ T cells cultured alone in complete medium and stimulated with 1 μg/ml anti-CD3 antibody. IFN-γ expression in T cells (H) and CD4^+^ T cells (I) was measured by FACS after co-culture for 24 h. All the data are presented as the means ± SD. n=4 for each group. *P<0.05, **P<0.01, ****P<0.0001. Other groups compared with the medium-only group: &&&P<0.001, &&&&P<0.0001. The data for the graphs (C), (G) and the groups of naïve B cells versus CTT B cells in the graph (H-I) were analyzed via two-tailed Student's t test; the data for the graphs (D-E) and the naïve MDSC (NM), CTT B-NM and CTT B-NM + iMHC-II groups in the graphs (H-I) were analyzed via one-way ANOVA.

To further investigate the underlying mechanisms by which B cells facilitate the maturation of MDSCs, MDSCs and B cells isolated from the spleen were labeled with CellTrace™ Far Red (red) and DiO (green), respectively, and co-cultured. Following a 20-hour co-culture period with B cells from the CTT group, a substantial quantity of green fluorescence was observed on the MDSCs, indicating that the membrane fractions of B cells had been engulfed by the MDSCs (**Figure 5A**). However, this phenomenon was rarely observed after co-culture with control B cells (Figure 5A). Indeed, the colocalization of red fluorescence with green fluorescence was markedly greater in B cells from the CTT group than in those from the control group (**Figure 5B-C**). Additionally, FACS analysis confirmed that the proportion of DiO^+^ MDSCs (gated on CD19^-^ CD11b^+^ Gr-1^+^ cells) was markedly greater in the CTT group than in the control group (**Figure 5D**). These findings indicate that CTT facilitates the engulfment of B-cell membrane components by MDSCs.

Polymorphonuclear leukocytes (PMNs) reportedly acquire MHC-II from B cells in lung tissue 26. Given the high expression of MHC-II on B cells and the increased uptake of B-cell membrane components by MDSCs following CTT, we hypothesized that the increased MHC-II on MDSCs following CTT was derived from B cells. To test this hypothesis, B cells from the control or CTT group were prelabeled with PE-conjugated anti-mouse IA/IE antibody and subsequently co-cultured with MDSCs from the control group for 20 hours. Notably, the acquisition of MHC-II molecules by MDSCs from B cells after CTT was markedly greater than that in the control group (**Figure 5E**). MHC-II molecules are only capable of exerting their biological function when presented at the surface of the cell membrane. Thus, the biotin-streptavidin system was employed to determine the location of MHC-II molecules derived from B cells following CTT. Specifically, B cells after CTT were prelabeled with biotinylated anti-mouse IA/IE antibody and co-cultured with MDSCs from the control group for 20 hours, and PE-streptavidin was used for cell surface staining of MDSCs (**Figure 5F**). Only the MHC-II-biotin complexes present on the cell surface can bind to PE-streptavidin. As shown in **Figure 5G**, the proportion of PE-conjugated streptavidin-positive MDSCs was notably greater in those co-cultured with biotin-labeled B cells from the CTT group than in those cocultured with cells from the control group. These results indicate that B-cell-derived MHC-II molecules can be integrated onto the membrane surface of MDSCs.

To determine whether the upregulation of MHC-II expression on MDSCs was driven predominantly by phagocytosis, we employed a dual-labeling strategy in which PE-conjugated streptavidin and PerCP/Cyanine5.5 anti-mouse IA/IE antibodies were used to label B-cell-derived MHC-II and endogenously synthesized MHC-II on MDSCs after co-cultured with biotinylated anti-mouse IA/IE antibody prelabeled B cells, respectively. As shown in **Figure S12**, the percentage of biotin⁺ MDSCs (Q1+Q2) was significantly greater than that of endogenous MHC-II⁺ MDSCs (Q2+Q3), which indicated that the phagocytosis of B-cell-derived MHC-II is the dominant contributor to the upregulated MHC II expression in MDSCs.

The mixed lymphocyte reaction (MLR), which is based on the recognition of allogeneic MHC molecules that are bound with peptides by T cells 27, is a widely employed method for the evaluation of T-cell alloreactivity. To confirm the biological effects of MHC-II molecules derived from B cells on MDSCs, MLR analysis was conducted. Splenic MDSCs from naïve BALB/c mice were co-cultured with splenic B cells from C57BL/6 mice (14 days after CTT) for 20 hours, after which these B cells were removed to obtain MDSCs that had taken up MHC-II from allogeneic B cells (stimulator cells). The acquisition of MHC-II molecules derived from B cells after CTT (C57BL/6 background) by naïve MDSCs from BALB/c mice was validated through FACS (**Figure S13**). These cells were subsequently co-cultured with naïve BALB/c mouse splenocytes (responder cells) for another 24 hours. As shown in **Figure 5H-I**, allogenic B cells (CTT B cells derived from C57BL/6 background mice) notably induced T-cell alloreactivity, as evidenced by the substantial increase in the proportions of IFN-γ^+^ T cells compared with those cultured with autologous B cells. Although autologous MDSCs were unable to elicit a T-cell response, MDSCs that had taken up MHC-II from allogeneic B cells obviously amplified the production of IFN-γ by T cells, an effect that was entirely abolished by the administration of the MHC-II blocking antibody (Figure 5H-I). These results indicate that MDSCs acquire functional MHC-II molecules derived from B cells following CTT.

### MDSCs integrate membrane components from B cells after CTT in a CD36-dependent manner

**Figure 6:**
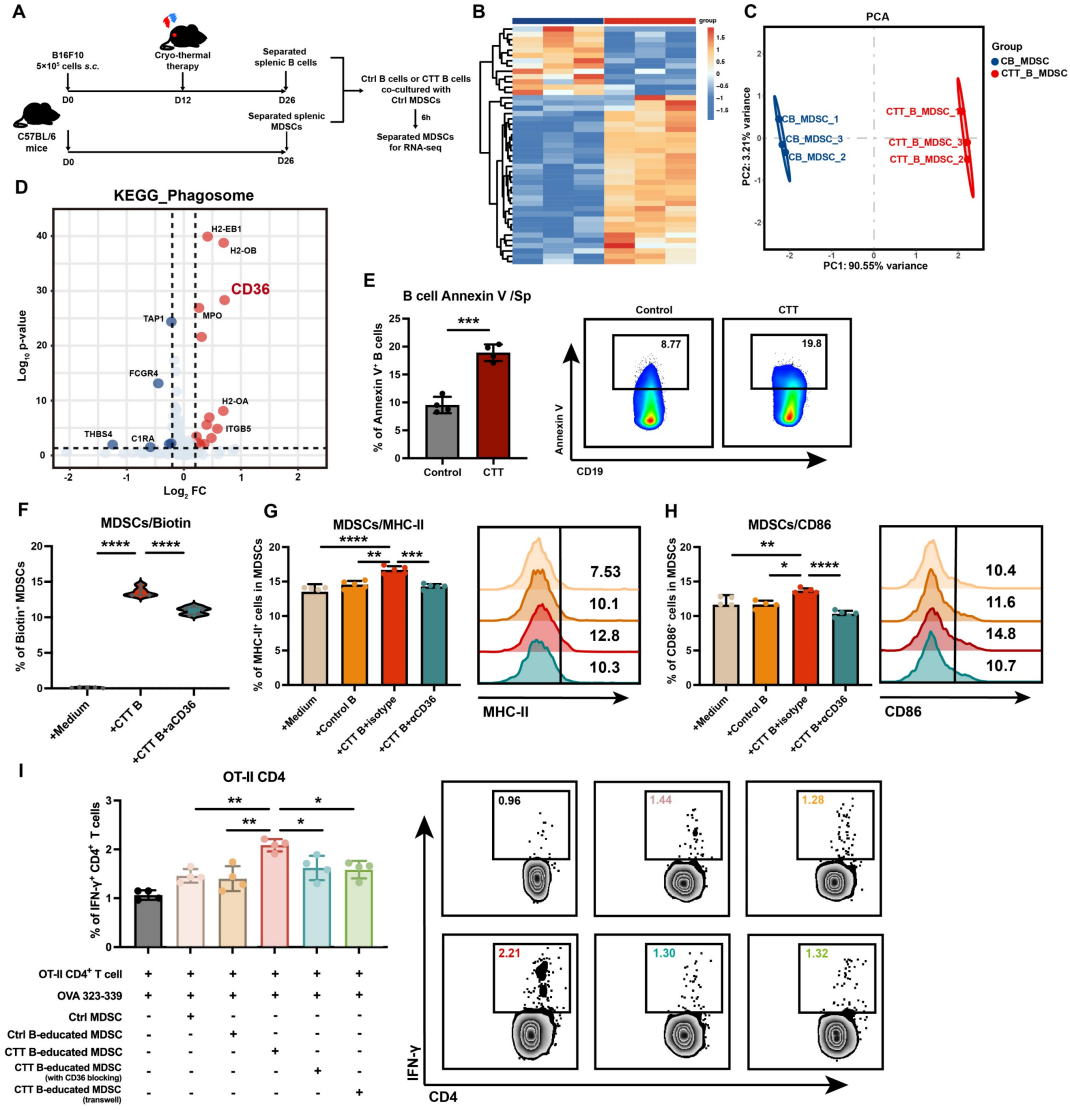
** MDSCs integrate B-cell-derived MHC-II in a CD36-dependent manner.** (A) Schematic of RNA sequencing of MDSCs after co-culture with B cells *in vitro*. (B) Heatmap of differentially expressed genes in MDSCs co-cultured with B cells from the control group and MDSCs co-cultured with B cells after CTT. (C) Principal component analysis (PCA) of MDSCs co-cultured with B cells from the control or CTT group. (D) Volcano map of the phagosome pathway from the KEGG database analyzed by GSEA. (E) The expression of Annexin V on splenic B cells 14 days after CTT. (F) The expression of biotin on MDSCs after CD36 blockade. Briefly, MDSCs from the control group were co-cultured with biotinylated anti-mouse IA/IE antibody-prelabeled B cells from the CTT group for 20 h (with or without 10 μg/ml anti-CD36 antibody), and streptavidin-PE was used to detect the biotin^+^ MDSCs. (G-H) The expression levels of MHC-II (G) and CD86 (H) in MDSCs co-cultured with B cells after CTT plus CD36 blockade. (I) IFN-γ expression in OT-II CD4^+^ T cells after co-culture with MDSCs in the presence of the OVA 323-339 peptide (10 µg/ml) for 18 h. MDSCs were cultured with medium, control B cells or CTT B cells with or without JC63.1 or in transwell chambers for 12 h. *P<0.05, **P<0.01, ***P<0.001, ****P<0.0001. Data for graph (E) were analyzed via two-tailed Student's t tests. Other groups compared with the medium-only group: &&&&P<0.0001. The data for graphs (F-I) were analyzed via one-way ANOVA.

To improve our understanding of the specific molecular mechanisms underlying B-cell-mediated MDSC maturation after CTT, we performed RNA sequencing on MDSCs co-cultured with either control B cells or CTT-induced B cells (**Figure 6A**). Compared with those co-cultured with control B cells, MDSCs co-cultured with CTT-induced B cells presented significantly different gene expression profiles (**Figure 6B, C**). Given the marked increase in membrane components from B cells engulfed by MDSCs after CTT, we focused on the expression of phagocytosis-related receptors on MDSCs. In the “phagosome” pathway from the KEGG database, CD36 was the most significantly upregulated molecule, in addition to MHC molecules (**Figure 6D**). Interestingly, in addition to the antigen processing and presentation pathway, the most significantly enriched gene cluster related to membrane signaling included CD36 (**Figure S14A, B**). As an apoptotic recognition receptor on phagocytes, CD36 binds to the phosphatidylserine (PS) exposed on the membranes of apoptotic cells, leading to efferocytosis and the regulation of inflammatory responses 28. Annexin V staining revealed that after CTT, the level of PS on the membrane of B cells was increased, which may facilitate the integration of B-cell components by MDSCs via CD36 binding (**Figure 6E**).

Therefore, we utilized an anti-CD36 monoclonal antibody (JC63.1) to block CD36 on MDSCs, followed by co-culture with B cells after CTT and subsequent flow cytometry analysis. The results indicated that anti-CD36 antibody blockade significantly reduced the percentage of biotin-positive MDSCs (**Figure 6F**). Moreover, the expression of MHC-II and CD86 on MDSCs co-cultured with B cells after CTT was completely abolished compared with that on those treated with an isotype control antibody (**Figure 6G, H**). Furthermore, the antigen-presenting capacity of MDSCs after incubation with CTT-induced B cells was significantly greater than that of MDSCs cultured alone or in the presence of control B cells (**Figure 6I**). However, this phenomenon was abrogated when CD36-blocking antibodies were utilized or transwell chambers were used to separate MDSCs from B cells after CTT (Figure 6I). These results demonstrated that MDSCs integrated functional MHC-II molecules from B cells after CTT in a CD36-dependent manner.

In conclusion, these findings demonstrate that after CTT, PS exposure on the membranes of B cells increases, and the binding between PS on B cells and CD36 on MDSCs facilitates the uptake and integration of B-cell membrane components by MDSCs, thereby promoting the antigen-presenting phenotype of MDSCs.

## Discussion

In this study, we demonstrated that activated B cells are crucial for inhibiting tumor metastasis, stimulating both innate and adaptive immune responses, and sustaining systemic long-term antitumor immunity in multiple tumor models. More importantly, our findings demonstrated that after CTT, splenic B cells inhibited the accumulation of MDSCs in the spleen and reprogrammed them into a mature, antigen-presenting phenotype by transferring MHC-II molecules to their surface via a CD36-dependent mechanism. This cascade facilitates the Th1-dominant differentiation of CD4^+^ T cells, resulting in durable systemic antitumor immunity.

Currently employed immunotherapy strategies predominantly target T cells or NK cells 29, often overlooking the diverse roles of B cells in reshaping the tumor microenvironment. B cells promote systemic antitumor immunity by enhancing the effector functions of CD4^+^ and CD8^+^ T cells in the spleen and lymph nodes, which leads to the inhibition of melanoma lung metastasis 30. Moreover, activated pulmonary B cells increase the killing capacity of NK cells by promoting IFN-γ production 31. In addition, the depletion of circulating B cells significantly contributes to the expansion of MDSCs in lymphatic tissues and tumor sites 32, underscoring the crucial role of B cells in regulating the TME 33. Our previous studies demonstrated that CTT, as a local ablative therapy, is capable of completely ablating *in situ* tumor tissue and inhibiting lung metastasis. Moreover, CTT has been found to induce robust and sustained CD4^+^ Th1-dominant systematic antitumor immunity in mouse tumor models and patients with colorectal cancer liver metastasis (CRCLM) 6, 9, 10, 34, 35. In this study, CTT activated splenic B cells, which inhibited tumor metastasis, promoted the maturation of MDSCs through CD36-dependent MHC-II transfer and subsequently stimulated the Th1-dominant T-cell immune response. These results broaden our understanding of the role of B cells in the CTT-mediated systemic antitumor immune response. Our findings indicate that the systemic activation of B cells represents a promising strategy for the development of more effective tumor immunotherapy strategies.

Activated intratumoral B cells act as antigen-presenting cells, directly promoting the Th1 differentiation of CD4^+^ T cells 36. By shaping the fate of tumor-specific CD4^+^ Tfh cells, tumor neoantigen-induced tumor-specific B cells increase the effector functions of CD8^+^ T cells 37. In this study, we confirmed that B cells in the effector phase (5 days after CTT) directly stimulated the differentiation of Th1 and Tfh cells and increased the expression of cytotoxic markers on CD8^+^ T cells *in vitro* (**Figure S15A, B**). However, at the late stage after CTT, B cells did not have a direct effect on CD4^+^ and CD8^+^ T-cell responses; instead, they affected the T-cell response by promoting the maturation of MDSCs. These findings emphasize that B cells mediate the T-cell-mediated antitumor immune response through specific regulatory pathways at different stages after CTT.

MDSCs are associated with the immunosuppressive tumor microenvironment, contributing to T-cell exhaustion and tumor immune evasion 38, which remain potential therapeutic targets. Direct depletion of MDSCs to alleviate tumor-mediated immunosuppression has been achieved in several studies 39, 40, and MDSC depletion is typically accompanied by accelerated accumulation of immature, regulatory MDSCs to compensate for their reduced numbers 41. Reprogramming MDSCs toward a mature phenotype via immunostimulants and drugs (such as ATRA, beta-glucan, and DHODH) can increase their immunostimulatory efficacy 42-45. Nevertheless, the mechanisms through which immune cell‒cell interactions facilitate the maturation of MDSCs remain poorly characterized. Our previous study revealed that CTT effectively promotes the maturation of MDSCs by stimulating NK cells to express NKG2D and secrete IFN-γ at the late stage after treatment 46. In this study, we revealed a novel mechanism by which CTT-induced B cells mediate the maturation of MDSCs through the transfer of MHC-II from B cells to MDSCs through the interaction of CD36. The interaction between B cells and MDSCs facilitates the maturation of MDSCs, which in turn promotes CD4^+^ Th1 differentiation and improves the effector function of CD8^+^ T cells after CTT. These findings highlight that CTT-induced systemic B-cell activation significantly promotes MDSC maturation and that CTT-triggered B-MDSC interactions play a crucial role in effective antitumor immunity.

DCs and B cells can integrate MHC-II molecules from other cells to increase their antigen-presenting capabilities in healthy or tumor-bearing mice 47, 48. This process depends on trogocytosis or exosome-mediated and cell‒cell contact-dependent cross-dressing 47-51. Moreover, CD8α^+^ DCs can acquire surface antigens from target cells during the development of T-cell tolerance in a CD36-dependent manner 52. Additionally, CD36 binds PS present on apoptotic cells or apoptotic bodies 53. Our present study revealed that CD36 is essential for the engulfment of B-cell-derived MHC-II molecules by MDSCs via direct cell‒cell contact in tumor models. After CTT, B cells presented elevated PS levels on their membrane surfaces, which could be attributed to the fact that after B-cell activation, the subsequent affinity selection process promoted the proliferation and differentiation of high-affinity B cells while simultaneously promoting the apoptosis of a different B-cell subset 54. Then, the activation-induced PS exposure on B-cell membranes led to increased interaction with MDSCs and facilitated the transfer of MHC-II molecules.

B-cell activation is initially driven by BCR engagement with antigens and can be amplified by pattern recognition receptors, such as TLRs, which reduce the activation threshold 55. In addition, cytokines such as BAFF, IL-21, and IFN-γ further increase B-cell activation and survival after BCR stimulation 56-58. Our previous studies provided evidence that CTT induces substantial release of tumor neoantigens 9 and damage-associated molecular patterns (DAMPs), including HSP70^5^ and DNA, which may directly stimulate BCR and TLR signaling, respectively. Furthermore, we observed increased secretion of IFN-γ by T cells and NK cells after CTT, accompanied by elevated expression of BAFF in MDSCs 59. Collectively, the above evidence indicates that CTT creates an environment conducive for B-cell activation, warranting further investigation into the underlying mechanisms involved.

B cells differentiate into plasma cells in response to specific antigens and secrete different isotypes of antibodies within lymphoid organs and tumor tissues. High levels of immunoglobulin are associated with cytotoxic CD8^+^ T-cell infiltration 60 and enhance the antibody-dependent cellular phagocytosis (ADCP) and antibody-dependent cellular cytotoxicity (ADCC) mediated by macrophages, DCs and NK cells 18, 61. In our present study, significant enrichment of the Notch signaling pathway, which is a crucial regulator of marginal zone formation, B-cell differentiation and antibody production, was observed in B cells after CTT 62. Further research is needed to investigate the dynamics and function of antibodies, especially tumor-specific antibodies, at different time points and stages after CTT, which would contribute to a better understanding of the role of B cells in modulating both innate and adaptive immunity.

In conclusion, our present study highlights the pivotal role of CTT-activated B cells in promoting the differentiation of MDSCs into mature antigen-presenting cells by facilitating the transfer of MHC-II molecules to the surface of immature MDSCs in a CD36-dependent manner. This immune-activating cascade is crucial for building CD4^+^ Th1-dominant systematic antitumor immunity and achieving effective therapeutic efficacy. Our findings elucidate the function of CTT-induced B cells, deepen our understanding of the mechanisms underlying CTT-induced immune activation, and reveal a novel cross-talk mechanism between B cells and MDSCs that could be used to develop new immunotherapy strategies.

## Methods

### Cell culture

The B16F10 melanoma cell line was generously donated by Professor Weihai Ying of the Med-X Research Institute, Shanghai Jiao Tong University. The 4T1 breast cancer cell line was provided by Shanghai First People's Hospital, China. B16F10, MC38 and 4T1 cells were cultured in Dulbecco's modified Eagle's medium (DMEM; Meilunbio, Dalian, China) supplemented with 10% fetal bovine serum (FBS; Gibco BRL, Rockville, MD, U.S.A.), 100 units mL^-1^ penicillin and 100 µg mL^-1^ streptomycin (HyClone, Logan, UT, USA). The cell cultures were maintained at 37°C in a humidified atmosphere containing 5% CO2.

### Animal models

Female BALB/c and C57BL/6 mice, aged between 6-8 weeks, were obtained from Shanghai Slaccas Experimental Animal Co., Ltd. (China). The mice were housed in isolated cages under a 12-hour light/dark cycle and provided sterile food and water maintained at a pH of 7.5-7.8. All the animal experiments adhered to the guidelines of the Animal Welfare Committee of Shanghai Jiao Tong University and were conducted in accordance with the protocols approved by the Shanghai Jiao Tong University Scientific Ethics Committee.

Tumor-bearing mice were generated by subcutaneously injecting approximately 5×10^5^ B16F10 cells into the right flank of C57BL/6 mice and 4×10^5^ 4T1 cells into the right flank of BALB/c mice. The MC38 bilateral tumor model was generated by subcutaneously injecting approximately 5×10^5^ cells into the right flank and 10^5^ cells into the left flank of C57BL/6 mice. Tumor-bearing mice were randomly assigned to cages and subjected to different treatments.

### Cryo-thermal therapy procedures

The cryo-thermal therapy system was developed in our laboratory. At approximately 12 days post tumor inoculation, when the tumor diameter reached 1 cm, the mice were randomly allocated to either the control (untreated-mice) group or the cryo-thermal therapy (CTT) group. Specifically, the mice in the CTT group were anesthetized and subjected to rapid freezing using liquid nitrogen followed by RF heating to achieve complete ablation of the primary tumor tissue. The details were described in our previous studies 9, 25.

### B-cell depletion

To deplete B cells, the mice in the CTT group received an intravenous injection of 250 μg of Ultra-LEAF™-purified anti-mouse CD20 antibody (SA271G2, BioLegend, San Diego, CA, USA) on day 5 after CTT.

### Statistical analysis

All experiments in this study were performed with at least two independent biological replicates. All the data are presented as the means ± standard deviations (SDs). Differences between two groups were determined by two-tailed Student's t tests. Comparisons between multiple groups were performed using one-way ANOVA, followed by Tukey's multiple comparisons test. The differences in tumor growth trends between groups were analyzed using 2-way ANOVA. GraphPad Prism 9.0 (La Jolla, CA) was used for all the statistical analyses.

## Supplementary Material

Supplementary figures and tables.
