# Supplementary material for: Activated B cells modulate the maturation of MDSCs via CD36-dependent MHC-II transfer to orchestrate CD4+ Th1-dominant antitumor immunity after cryo-thermal therapy
Source: Int J Biol Sci. 2025 Aug 30;21(12):5547–62. doi: 10.7150/ijbs.115232 (PMC12435582; doi:10.7150/ijbs.115232)
Supplement: Supplementary file 1 — Supplementary figures and tables. [file ijbsv21p5547s1.pdf]

Supporting Information

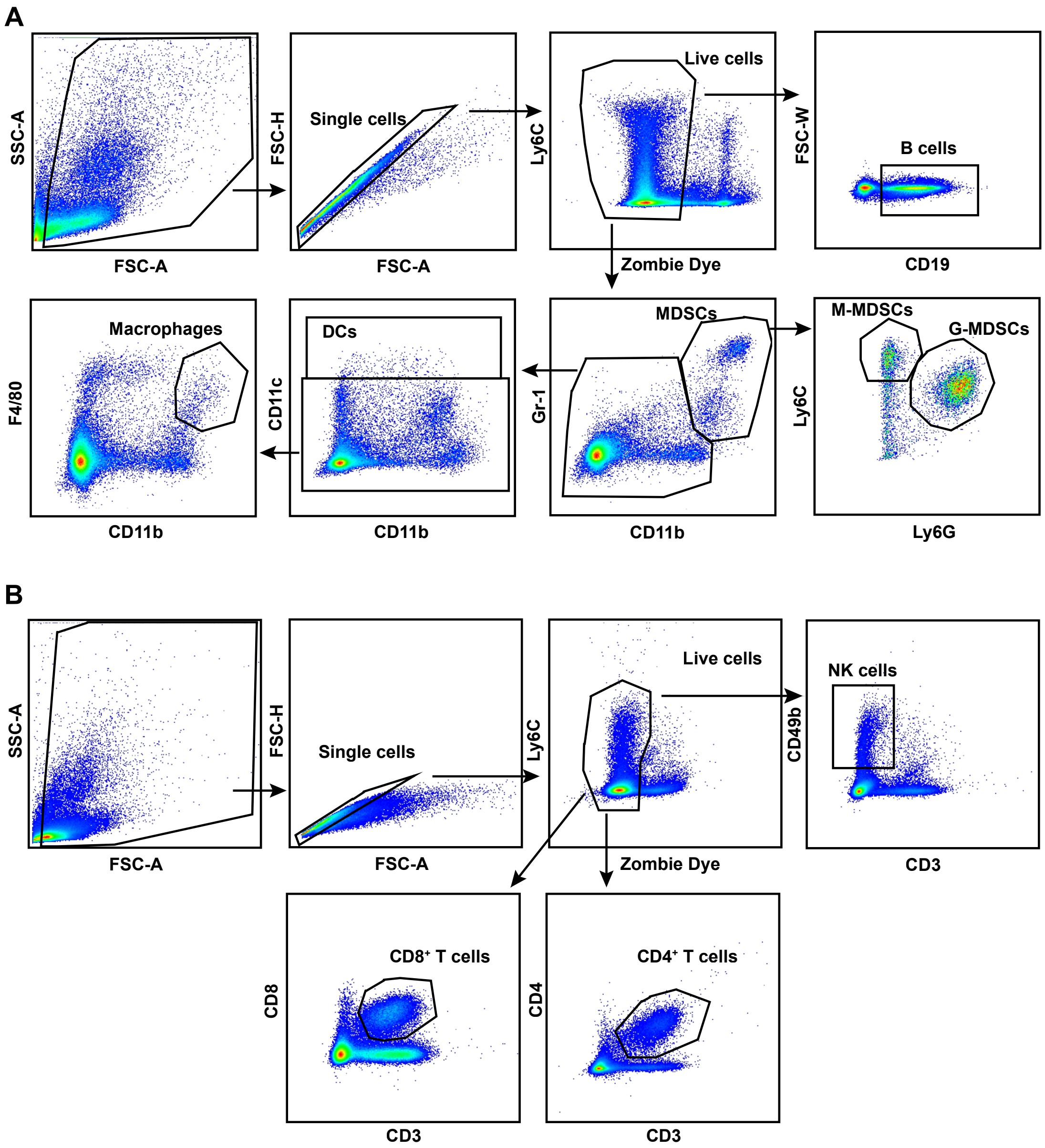

Figure S1. Gating strategy for flow cytometry. (A)Gating strategy of MDSCs, DCs, Macrophages, and B cells. (B) Gating strategy of T cells and NK cells.

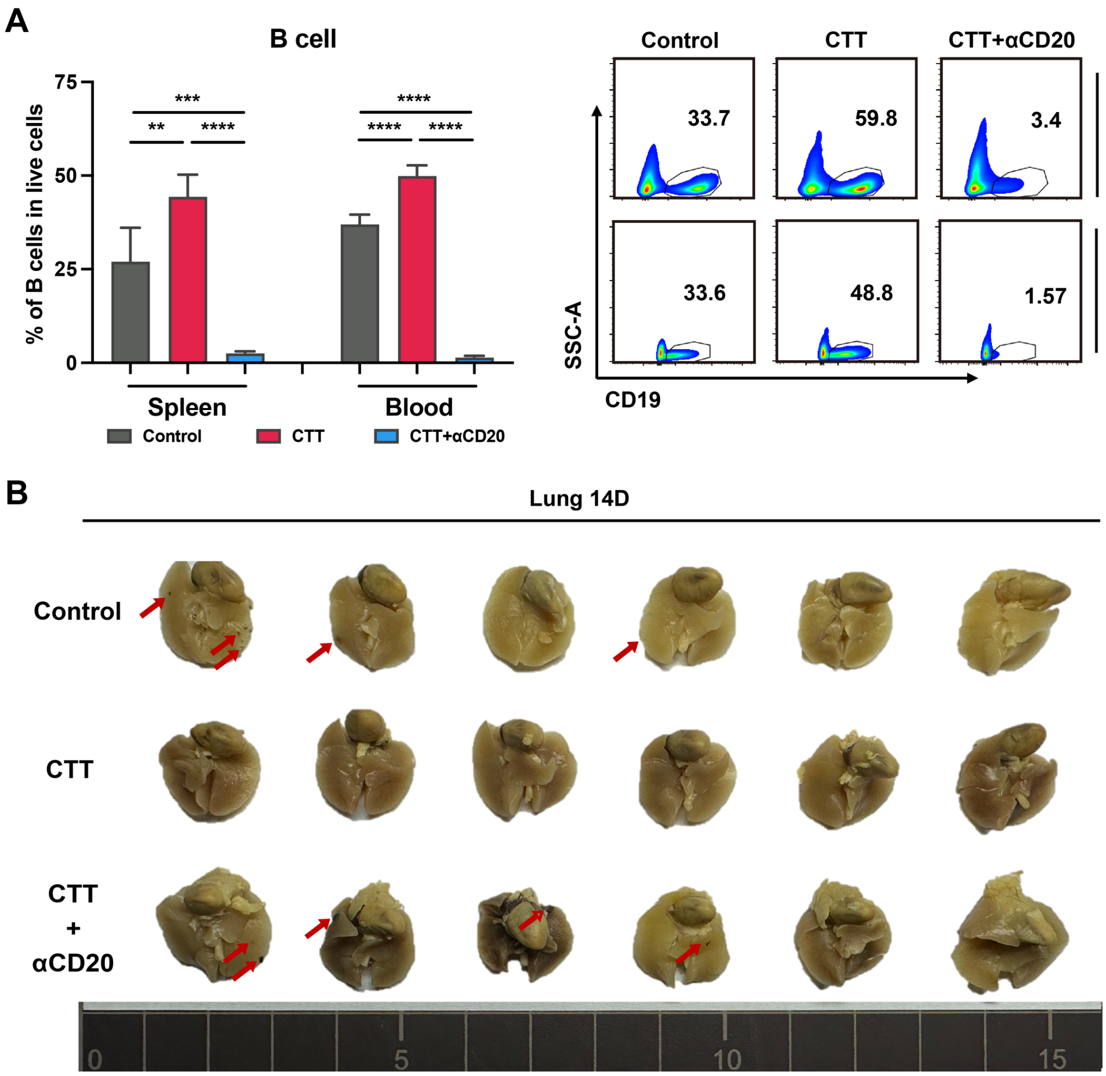

Figure S2. B cells were successfully depleted by anti-CD20 monoclonal antibody. (A) Bar plots and FACS representative plots of B cells from the control, CTT and CTT plus B-cell depletion groups on day 14 after CTT. (B) The lungs were fixed with paraformaldehyde and collected from different groups on day 14 after CTT. All the data are presented as the means ± SD. n=4 for each group. **P<0.01, ***P<0.001, ****P<0.0001. The data in panel (A) were analyzed via one-way ANOVA.

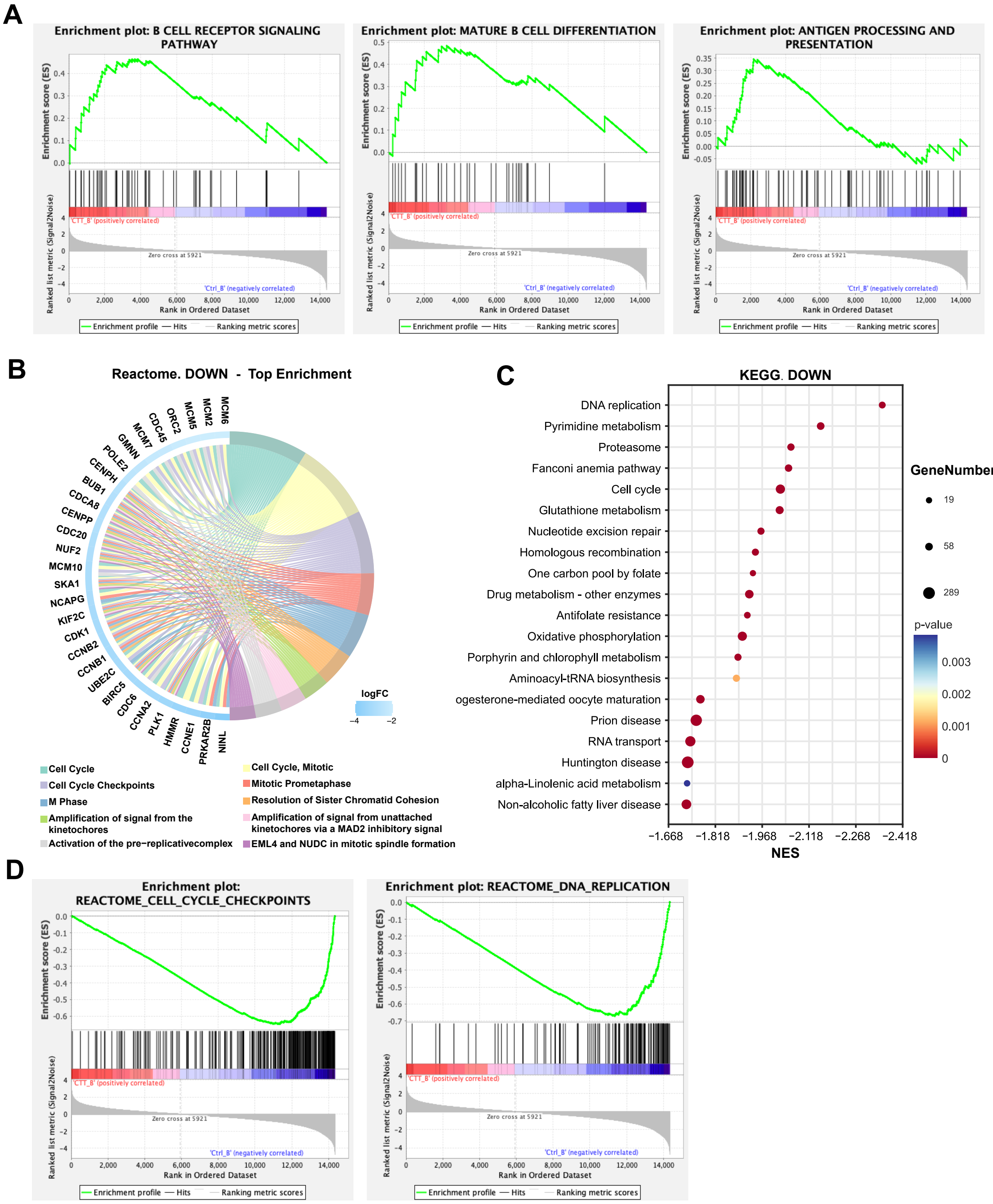

Figure S3. CTT enriched pathways related to antigen processing and presentation and activation and downregulated those related to the cell cycle and DNA replication pathways in B cells. (A) GO term GSEA (B cells from the CTT group versus B cells from the control group) of B-cell receptor signaling, mature B-cell differentiation and antigen processing and presentation. (B) Core network with the top 10 Reactome pathways identified via enrichment analysis. (C) Scatter plot of the top 20 downregulated KEGG pathways according to GSEA. (D) Gene set enrichment analysis (GSEA) results (B cells from the CTT

group versus B cells from the control group) of the cell cycle checkpoint and DNA replication terms.

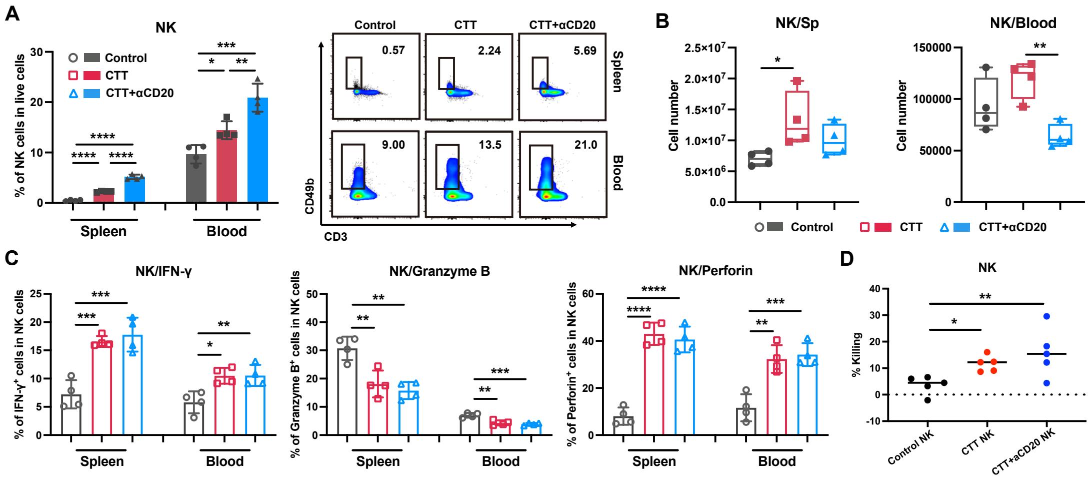

Figure S4. The numbers, effective markers and cytotoxic ability of NK cells on day 14 after CTT with B-cell depletion. (A-B) The percentages (A) and precise numbers (B) of NK cells in the spleen and blood. (C) The expression of IFN-γ, perforin and granzyme B on NK cells in the spleen and blood. (D) Killing assay of NK cells; E:T = 8:1. All the data are presented as the means ± SD. In panel (A-C), n=4 for each group; in panel (D), n=5 for each group. *P<0.05, **P<0.01, ***P<0.001, ****P<0.0001. The data for the graphs were analyzed via one-way ANOVA.

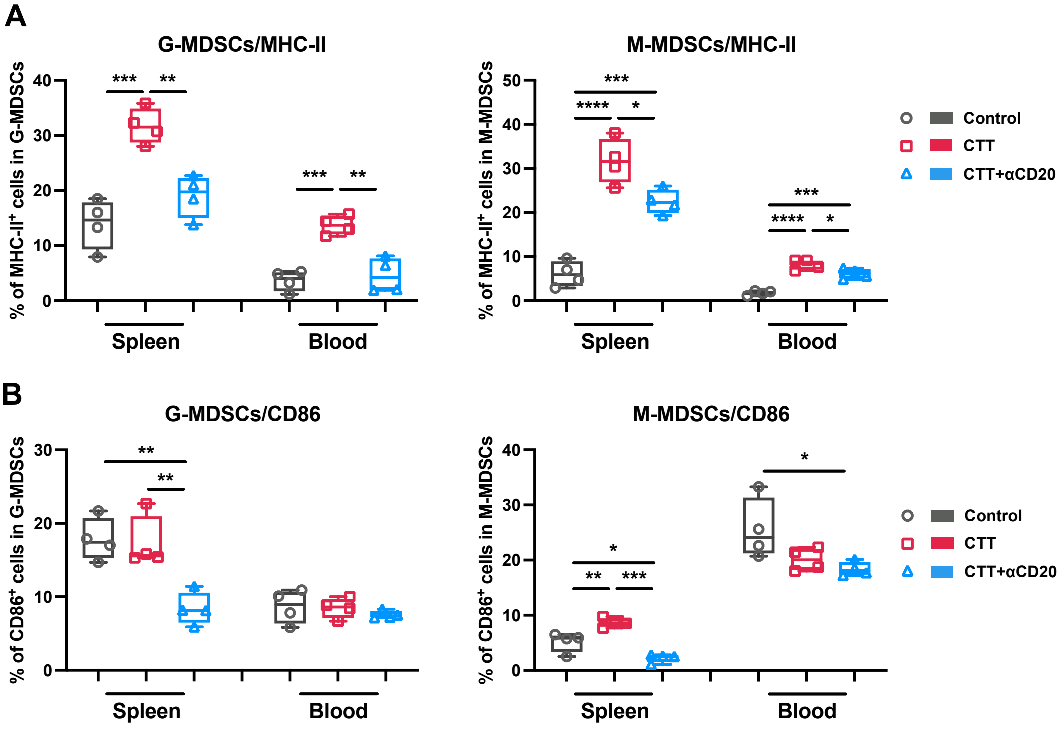

Figure S5. B cells are essential for the maturation of G-/M-MDSCs induced by CTT. (A-B) The percentage expression of MHC-II (A) and CD86 (B) molecules in G-/M-MDSCs (*in vivo*) on day 14 after CTT. All the data are presented as the means ± SD. n=4 for each group. *P<0.05, **P<0.01, ***P<0.001, ****P<0.0001. The data for the graphs were analyzed via one-way ANOVA.

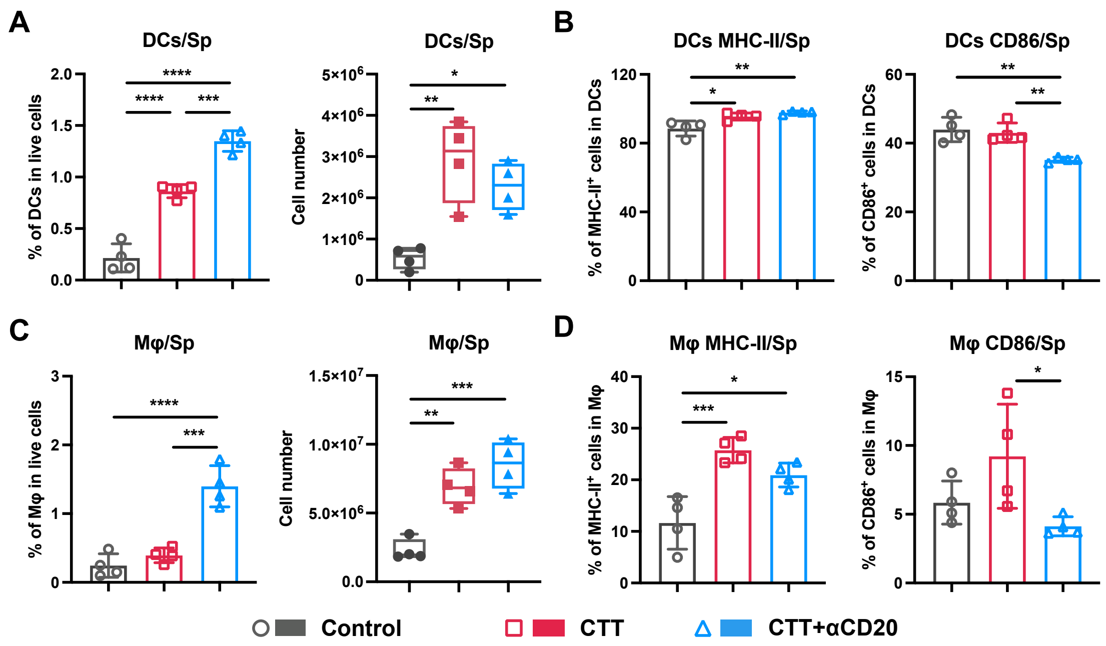

Figure S6. The numbers and phenotypes of DCs and macrophages on day 14 after CTT with B-cell depletion. (A) The percentage and absolute number of DCs. (B) Percent expression of MHC-II and CD86 molecules on DCs. (C) The percentage and precise number of macrophages. (D) Percentages of MHC-II- and CD86-expressing macrophages. All the data are presented as the means ± SD. n=4 for each group. *P<0.05, **P<0.01, ***P<0.001, ****P<0.0001. The data for the graphs were analyzed via one-way ANOVA.

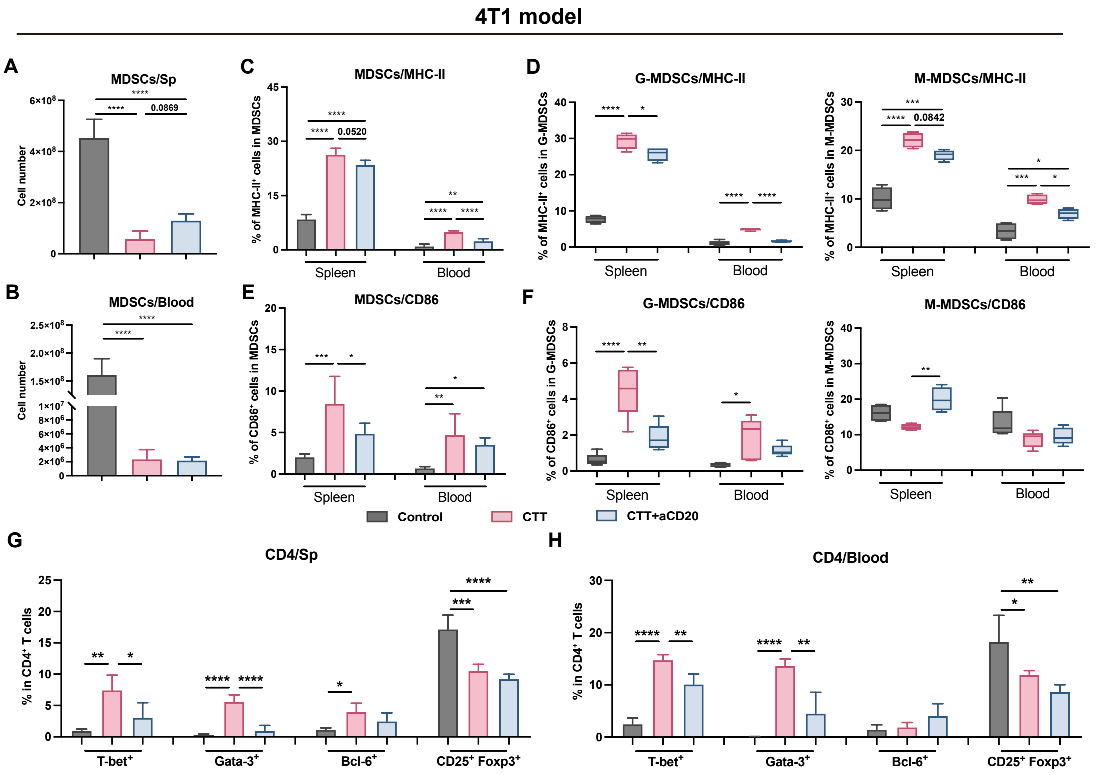

Figure S7. The effect of B-cell depletion after CTT in 4T1 model mice. 4T1 tumor-bearing mice were treated with CTT on day 16 after transplantation. B cells were depleted *in vivo* on day 5 after CTT, and the changes in immune cells were analyzed on day 21 after CTT. (A-B) The numbers of MDSCs in the spleen (A) and blood (B) 14 days after CTT in the 4T1 model. (C-D) The expression of MHC-II on MDSCs (C) and G-/M-MDSCs (D). (E-F) The percentage of CD86-expressing MDSCs (E) and G-/M-MDSCs (F). (G-H) Subsets of $CD4^+$ T cells in the spleen (G) and blood (H). All the data are presented as the means ± SD. n=4 for each group. *P<0.05, **P<0.01, ***P<0.001, ****P<0.0001. The data for the graphs were analyzed via one-way ANOVA.

MC38 model

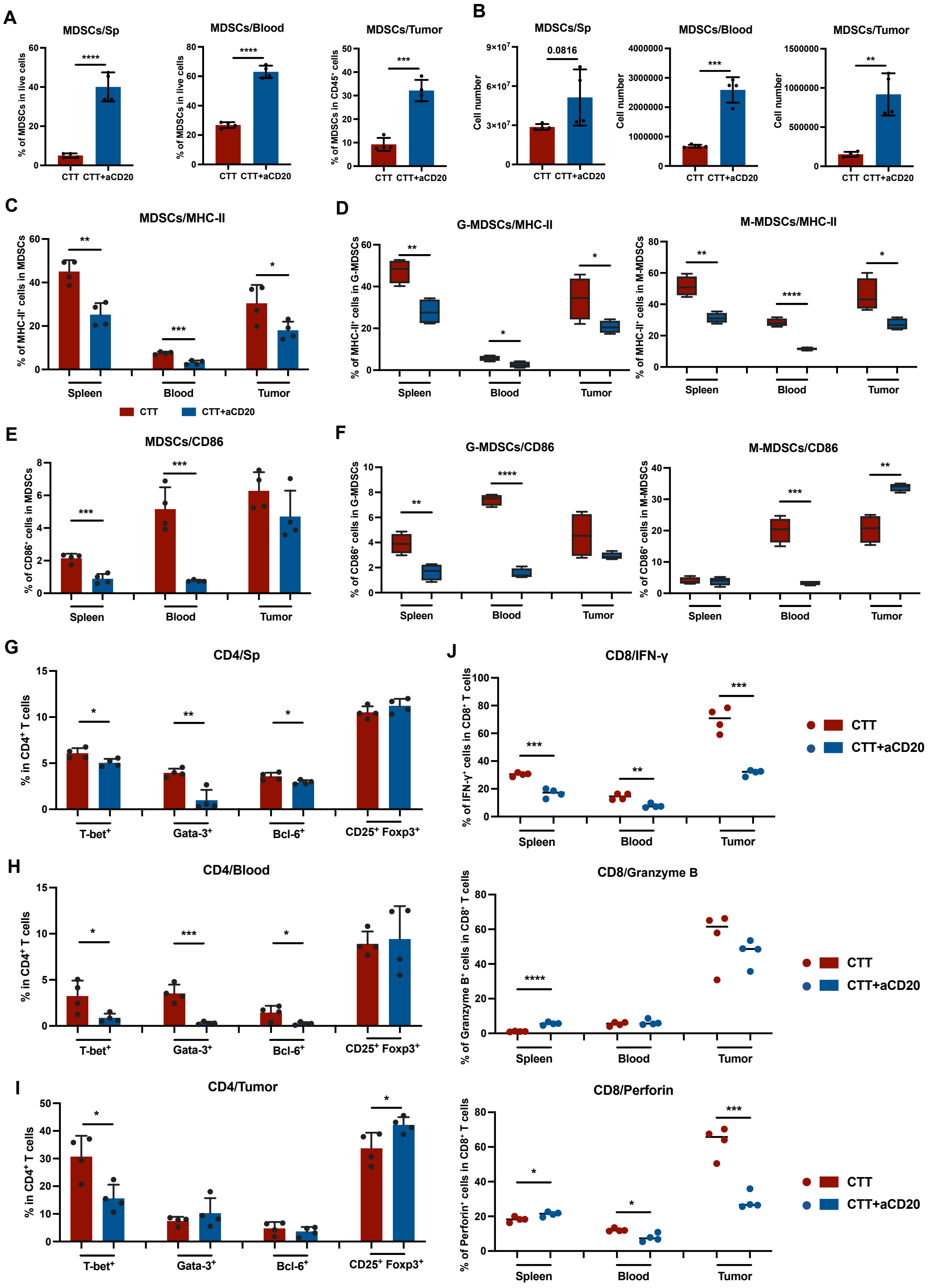

Figure S8. The effect of B-cell depletion after CTT in MC38 model mice. MC38 tumor-bearing mice were treated (right-sided tumors) with CTT on day 12 after transplantation. B cells were depleted *in vivo* on day 5 after CTT, and the changes in immune cells were analyzed on day 20 after CTT. (A) The percentages of MDSCs in the spleen, blood and left

tumor. (B) The numbers of MDSCs in the spleen, blood and left tumor. (C-D) The expression of MHC-II on MDSCs (C) and G-/M-MDSCs (D). (E-F) The percentage of CD86-expressing MDSCs (E) and G-/M-MDSCs (F). (G-I) Subsets of $CD4^+$ T cells in the spleen (G), blood (H) and left tumor (I). (J) IFN-γ, perforin and granzyme-B expression on $CD8^+$ T cells in the spleen, blood and tumor. All the data are presented as the means ± SD. n=4 for each group. *P<0.05, **P<0.01, ***P<0.001, ****P<0.0001. The data for the graphs were analyzed via two-tailed Student's t tests.

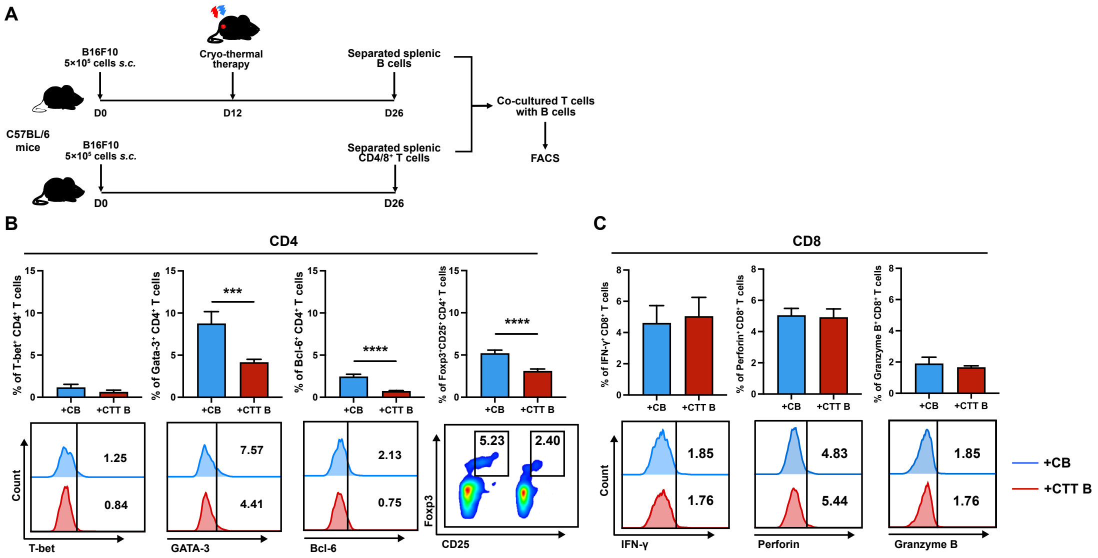

Figure S9. B cells isolated on day 14 after CTT did not directly promote the differentiation of CD4$^+$ T cells or the effector function of CD8$^+$ CTLs. (A) Scheme of B cells cocultured with control group T cells *in vitro*. (B-C) CD4$^+$ T-cell subsets (B) and the expression of effector molecules in CD8$^+$ T cells (C) cocultured with B cells from the control group (CB) or CTT group (CTT B) *in vitro*. The corresponding mouse serum was added to mimic the *in vivo* cytokine environment. All the data are presented as the means ± SD. n=4 for each group. ***P<0.001, ****P<0.0001. The data for the graphs were analyzed via a two-tailed Student's t test.

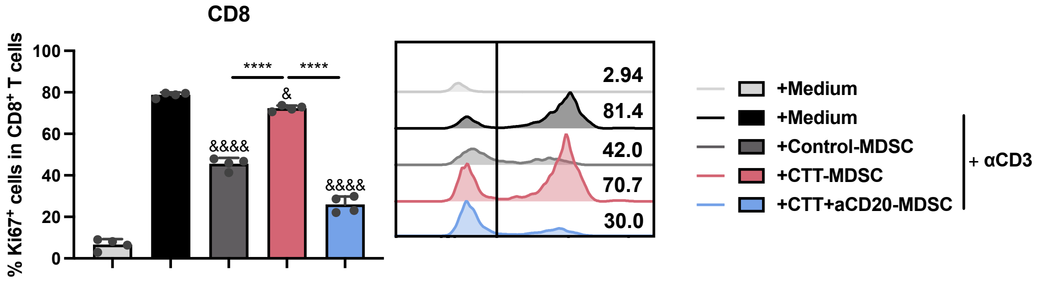

Figure S10. The proliferation of CD8^{+} T cells after cocultured with MDSCs from different groups. The Ki67 expression of CD8^{+} T cells cultured with MDSCs from the control, CTT or CTT with anti-CD20 groups was analyzed after 72 h; E:T = 2:1. All the data are presented as the means ± SD. N=4 for each group. ****P<0.0001. Other groups compared with the medium plus αCD3 group: &P<0.05, &&&&P<0.0001. The data for the graphs were analyzed via one-way ANOVA.

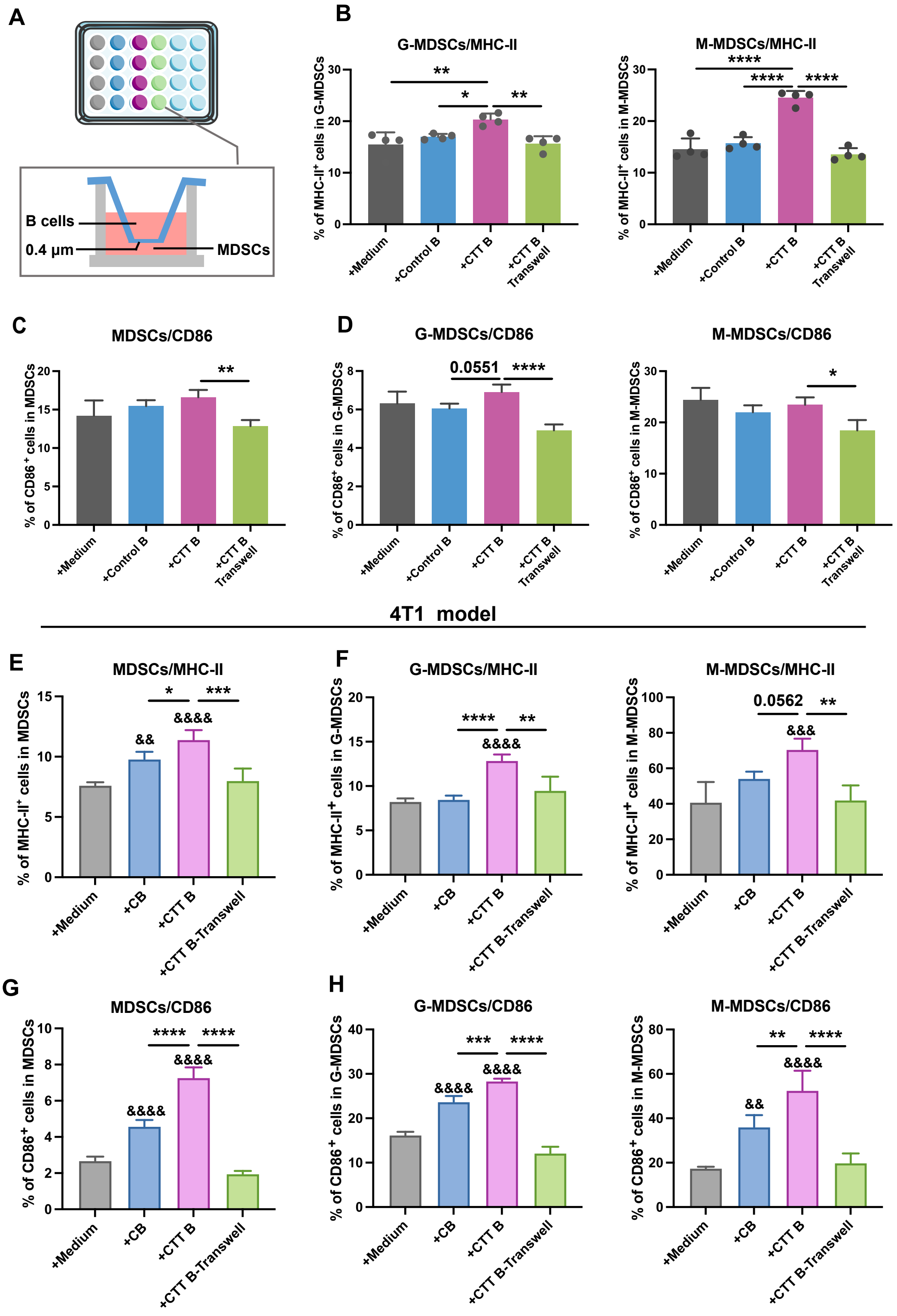

Figure S11. The expression of maturation molecules on MDSCs. (A) Schematic diagram of the transwell assay. B cells from the CTT group on day 14 after CTT were cultured in transwell chambers with 0.4 μm pores, and MDSCs from the control group were placed in the 24 wells under the chambers for 20 h and then analyzed by FACS. (B) The expression of

MHC-II molecules on G-MDSCs and M-MDSCs. (C) The expression of the CD86 molecule on MDSCs in the B16F10 model. (D) The expression of the CD86 molecule on M-MDSCs and G-MDSCs in the B16F10 model. (E) The expression of MHC-II molecules on MDSCs in the 4T1 model. (F) The expression of MHC-II molecules on M-MDSCs and G-MDSCs in the 4T1 model. (G) The expression of the CD86 molecule on MDSCs in the 4T1 model. (H) The expression of the CD86 molecule on M-MDSCs and G-MDSCs in the 4T1 model. All the data are presented as the means ± SD. n=4 for each group. *P<0.05, **P<0.01, ***P<0.001, ****P<0.0001. Other groups compared with the medium-only group: &&P<0.01, &&&&P<0.0001. The data for the graphs were analyzed via one-way ANOVA.

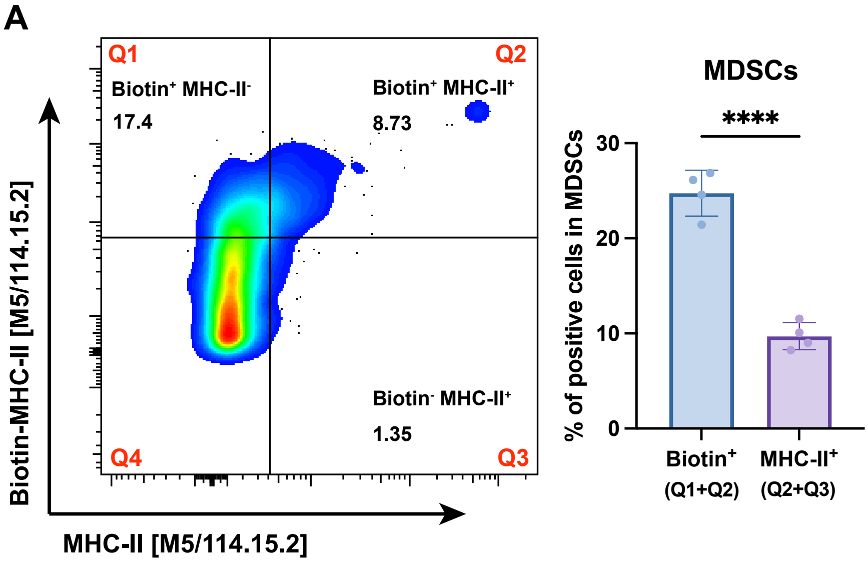

Figure S12. The expression of biotin-conjugated MHC-II and endogenous MHC-II on MDSCs after co-cultured with B cells after CTT. Q1 represents the population of only phagocytosed (B-cell-derived) MHC-II positive MDSCs; Q3 represents the population of only endogenously synthesized MHC-II positive MDSCs; while Q2 represents the population of both phagocytosed and endogenous MHC-II positive MDSCs.

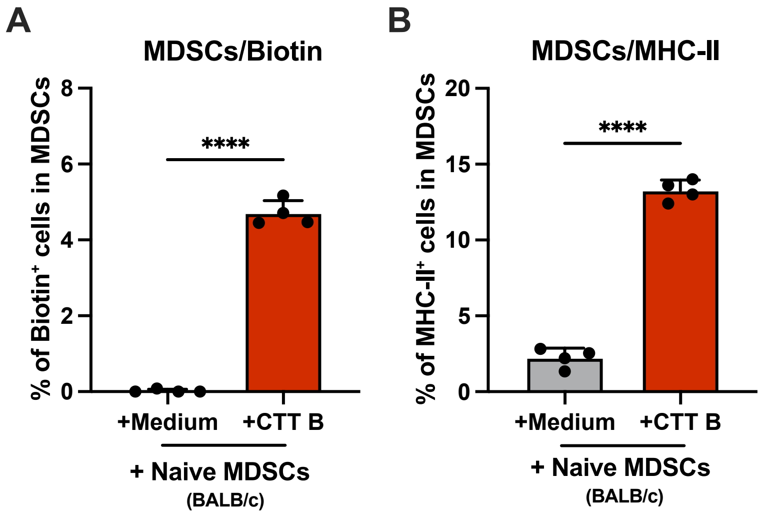

Figure S13. The expression of biotin and MHC-II on naïve MDSCs from BALB/c mice after coculture with B cells after CTT from C57BL/6 mice. (A) Expression of biotin on naïve MDSCs. (B) The expression of MHC-II molecules on naïve MDSCs. ****P<0.0001. The data for all the graphs were analyzed via two-tailed Student's t tests.

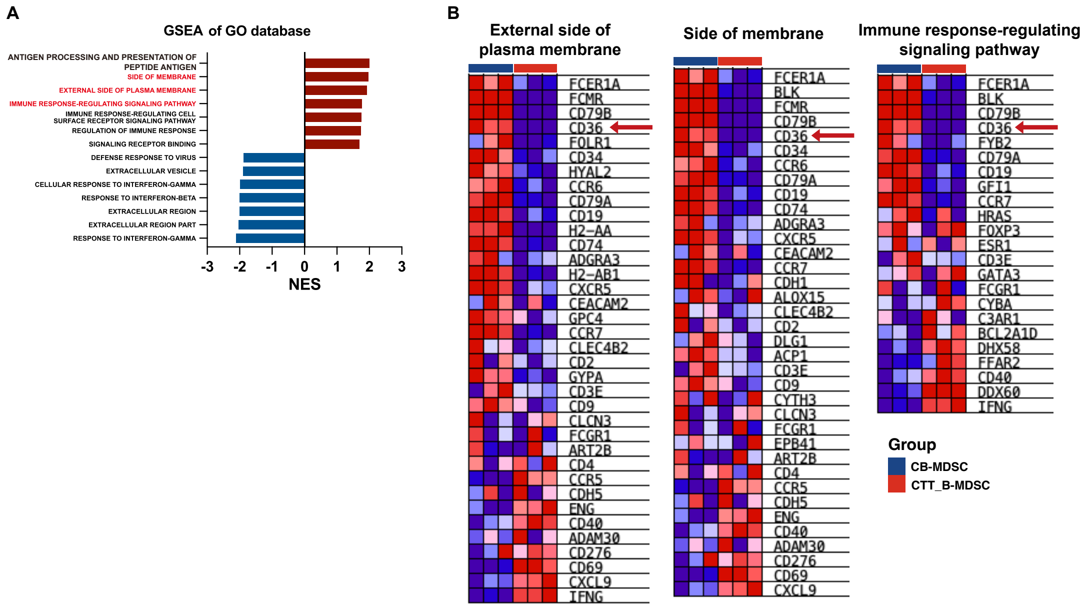

Figure S14. GSEA of the membrane signaling-related pathways of MDSCs. (A) Pathways enriched in MDSCs cocultured with CTT B cells compared with those enriched in those cocultured with control B cells. (B) Heatmap of gene expression in specific pathways showing the significant enrichment of CD36 in MDSCs cocultured with B cells after CTT.

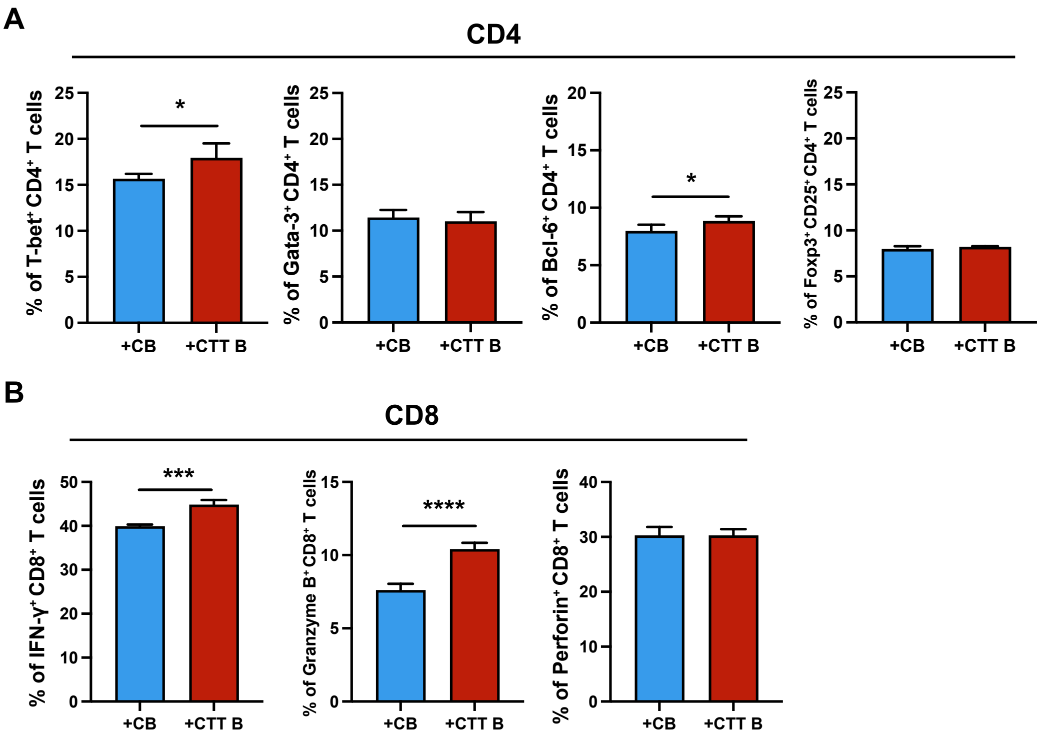

Figure S15. Activated B cells isolated on day 5 after CTT directly promote the differentiation of Th1 and Tfh CD4$^+$ T cells and the effector function of CD8 CTL. (A-B) The CD4$^+$ T cells subsets (A) and the effector molecules expression of CD8$^+$ T cells (B) co-cultured with B cells from the control group (CB) or CTT group (CTT B) *in vitro*. Corresponding mice serum was added to mimic the in vivo cytokine environment. All data were shown as mean ± SD. n=4 for each group. *P<0.05, ***P<0.001, ****P<0.0001. Data for graphs were calculated via two-tailed Student's T-test.

Table S1. Flow cytometry antibodies used in this study

Antibodies	Clone	Source	Cat#
Pacific Blue anti-mouse CD11b	M1/70	BioLegend	101224
APC anti-mouse Gr-1	RB6-8C5	BioLegend	108412
FITC anti-mouse Ly-6C	HK1.4	BioLegend	128006
PE/Cyanine7 anti-mouse Ly-6G	1A8	BioLegend	127618
APC anti-mouse Ly-6G	1A8	BioLegend	127614
PE anti-mouse CD11c	N418	BioLegend	117306
Brilliant Violet 711™ anti-mouse F4/80	BM8	BioLegend	123147
APC/Cyanine7 anti-mouse CD86	GL-1	BioLegend	105030
PE anti-mouse $H\text{-}2K^b/H\text{-}2D^b$	28-8-6	BioLegend	114607
PerCP/Cyanine5.5 anti-mouse I-A/I-E	M5/114.15.2	BioLegend	107626
BUV395 Rat Anti-Mouse CD40	3/23	BD Biosciences	745697
Alexa Fluor® 700 anti-mouse CD19	6D5	BioLegend	115528
PerCP/Cyanine5.5 anti-mouse CD3ε	145-2C11	BioLegend	100328
FITC anti-mouse CD3ε	145-2C11	BioLegend	100306
APC/Cyanine7 anti-mouse CD3ε	145-2C11	BioLegend	100330
PE/Cyanine7 anti-mouse CD4	RM4-5	BD Biosciences	552775
APC anti-mouse CD4	RM4-5	BD Biosciences	561091
Pacific Blue anti-mouse CD8a	53-6.7	BioLegend	100725
Alexa Fluor® 700 anti-mouse CD8a	53-6.7	BioLegend	100730
PerCP/Cyanine5.5 anti-mouse CD49b	DX5	BioLegend	108916
PE/Cyanine7 anti-mouse CD25	3C7	BioLegend	101916
PE anti-mouse Foxp3	MF-14	BioLegend	126404
Brilliant Violet 605™ anti-T-bet	4B10	BioLegend	644817

Alexa Fluor® 488 anti-GATA3	16E10A23	BioLegend	653808
BV421 Mouse Anti-Bcl-6	K112-91	BD Biosciences	563363
Brilliant Violet 605™ anti-mouse IFN-γ	XMG1.2	BioLegend	505840
Alexa Fluor® 647 anti-human/mouse Granzyme B	GB11	BioLegend	515406
PE anti-mouse Perforin	S16009A	BioLegend	154306
BUV395 Mouse Anti-Ki-67	B56	BD Biosciences	564071
Biotin anti-mouse I-A/I-E	M5/114.15.2	BioLegend	107603
PE Streptavidin		BioLegend	405203

Table. S2 Primer sequences of genes in this study

Name	Primer Sequence (5´-3´)
IL-1β-F	ACAGCAGCACATCAACAAGAG
IL-1β-R	ATGGGAACGTCACACACCAG
IL-10-F	GCTCTTACTGACTGGCATGAG
IL-10-R	CGCAGCTCTAGGAGCATGTG
Ebi3-F	CGGTGCCCTACATGCTAAAT
Ebi3-R	GCGGAGTCGGTACTTGAGAG
TGF-β-F	CTCCCGTGGCTTCTAGTGC
TGF-β-R	GCCTTAGTTTGGACAGGATCTG
IL-12-F	TGGTTTGCCATCGTTTTGCTG
IL-12-R	ACAGGTGAGGTTCACTGTTTCT
IFN-β-F	CTCCCACGTCAATCTTTCCTC
IFN-β-R	TGGGTGGAATGAGACTATTGTTG
IFN-γ-F	ATGAACGCTACACACTGCATC
IFN-γ-R	CCATCCTTTTGCCAGTTCCTC
TNF-α-F	TTCTGTCTACTGAACTTCGGGGTGATCGGTCC
TNF-α-R	GTATGAGATAGCAAATCGGCTGACGGTGTGGG
IL-6-F	GACAAAGCCAGAGTCCTTCAGAGAGATACAG
IL-6-R	TTGGATGGTCTTGGTCCTTAGCCAC
Granzyme B-F	CCACTCTCGACCCTACATGG
Granzyme B-R	GGCCCCCAAAGTGACATTTATT
TLR2-F	GCAAACGCTGTTCTGCTCAG
TLR2-R	AGGCGTCTCCCTCTATTGTATT
TLR4-F	ATGGCATGGCTTACACCACC
TLR4-R	GAGGCCAATTTTGTCTCCACA
TLR-7-F	ATGTGGACACGGAAGAGACAA
TLR7-R	GGTAAGGGTAAGATTGGTGGTG
TLR9-F	ACAACTCTGACTTCGTCCACC

TLR9-R	TCTGGGCTCAATGGTCATGTG
CD40-F	TGTCATCTGTGAAAAGGTGGTC
CD40-R	ACTGGAGCAGCGGTGTTATG
CD38-F	TCTCTAGGAAAGCCCAGATCG
CD38-R	GTCCACACCAGGAGTGAGC
ICOSL-F	AGCTCCATGTTTCTAGCGGGTTC
ICOSL-R	ACCATTGCACCGACTTCAGTCTC
CR2-F	AACACATGGTTACCAGGTGTACC
CR2-R	CGTGCCTCTCCAGCCATAAG
TIM4-F	CTACAGACATAGCCGTACTCA
TIM4-R	GTCTTCATCATCCCTCCC
PD-1-F	ACCCTGGTCATTCACTTGGG
PD-1-R	CATTTGCTCCCTCTGACACTG
